# Crucial role of the NSE1 RING domain in Smc5/6 stability and FANCM-independent fork progression

**DOI:** 10.1007/s00018-024-05275-3

**Published:** 2024-06-07

**Authors:** Neus P Lorite, Sonia Apostolova, Marta Guasch-Vallés, Aaron Pryer, Fernando Unzueta, Raimundo Freire, Roger Solé-Soler, Neus Pedraza, Xavier Dolcet, Eloi Garí, Neus Agell, Elaine M Taylor, Neus Colomina, Jordi Torres-Rosell

**Affiliations:** 1 Departament de Ciències Mèdiques Bàsiques, Institut de Recerca Biomèdica de Lleida, Universitat de Lleida, Lleida, 25198 Spain; 2 Lancaster Medical School, Faculty of Health and Medicine, Lancaster University, Lancaster, UK; 3 Departament Biomedicina, Universitat de Barcelona, Institut d’Investigacions Biomèdiques August Pi i Sunyer (IDIBAPS), Barcelona, Spain; 4 Unidad de Investigación, Hospital Universitario de Canarias, La Laguna, Santa Cruz de Tenerife, Spain; 5 Instituto de Tecnologías Biomédicas, Centro de Investigaciones Biomédicas de Canarias, Facultad de Medicina, Universidad de La Laguna, Campus Ciencias de la Salud, Santa Cruz de Tenerife, Spain; 6 Universidad Fernando Pessoa Canarias, Las Palmas de Gran Canaria, Spain; 7 Present Address: Institute of Biophysics and Biomedical Engineering, Bulgarian Academy of Sciences, Sofia, Bulgaria

**Keywords:** DNA replication, Genomic stability, NSE1, Smc5/6, SMC5, SMC6, NSE2, NSE3, NSE4, FANCM, RING, DNA fiber, MMS, Anaphase, Fanconi anemia

## Abstract

The Smc5/6 complex is a highly conserved molecular machine involved in the maintenance of genome integrity. While its functions largely depend on restraining the fork remodeling activity of Mph1 in yeast, the presence of an analogous Smc5/6-FANCM regulation in humans remains unknown. We generated human cell lines harboring mutations in the NSE1 subunit of the Smc5/6 complex. Point mutations or truncations in the RING domain of NSE1 result in drastically reduced Smc5/6 protein levels, with differential contribution of the two zinc-coordinating centers in the RING. In addition, *nse1-RING* mutant cells display cell growth defects, reduced replication fork rates, and increased genomic instability. Notably, our findings uncover a synthetic sick interaction between Smc5/6 and FANCM and show that Smc5/6 controls fork progression and chromosome disjunction in a FANCM-independent manner. Overall, our study demonstrates that the NSE1 RING domain plays vital roles in Smc5/6 complex stability and fork progression through pathways that are not evolutionary conserved.

**Supplementary Information:**

The online version contains supplementary material available at 10.1007/s00018-024-05275-3.

## Introduction

The efficient progression of DNA replication forks is of high importance to complete chromosome duplication and maintain genome integrity in dividing cells. Numerous players, including DNA polymerases, helicases, DNA translocases and topoisomerases, work synergistically to ensure the advancement of replication forks. In addition, different signaling pathways, such as the replicative stress and DNA damage checkpoints, contribute to maintaining genome integrity during DNA replication. Importantly, perturbations in these processes have been implicated in a wide range of human diseases, mostly characterized by genomic instability, including cancer [1].

Smc5/6 is an evolutionarily conserved multi-subunit complex and plays vital roles in genome stability [2]. Smc5/6 is a member of the eukaryotic SMC family of protein complexes, which also includes cohesin and condensin. Collectively, SMC complexes organize chromatin fibers to facilitate various chromosomal transactions, including gene expression, DNA replication, DNA repair and chromosome segregation. All SMC complexes present an ATPase activity that, in coordination with a series of conformational changes in the SMC molecule, promotes the progressive extrusion of loops of DNA. This activity is central for the ability of SMC complexes to organize chromatin fibers and, ultimately, chromosomes [3].

The Smc5/6 complex has an elongated structure, with two ATPase heads at one end connected through an arm domain to a hinge dimerization domain at the other end [4]. The Smc5/6 complex, in addition to the core SMC proteins SMC5 and SMC6, associates with six additional non-SMC subunits (NSE or non-SMC elements), most of which bind near the ATPase heads. NSE4 belongs to the kleisin family of SMC subunits and connects the SMC5 ATPase to the neck region in the arm domain of SMC6 [4–6]. In addition, NSE4 associates with the NSE1 and NSE3 subunits [7]. NSE1 contains a C-terminal RING domain with E3 ubiquitin ligase activity [8, 9]. In yeast, Nse1 promotes ubiquitination of various targets involved in cell metabolism and ribosome biogenesis [10]. The NSE3 subunit, also known as MAGE-G1, is related to the melanoma-associated antigen (MAGE) protein family and participates in binding to DNA [11]. The SLF1-SLF2 (Nse5-Nse6) heterodimer interacts with the ATPase heads and the head proximal arm domains and helps to coordinate the ATPase with the DNA-association and loop extrusion activities of the Smc5/6 complex [12–14]. Finally, the NSE2 subunit associates with the SMC5 arm through its N-terminal domain, an interaction that is essential for cell viability [15, 16]. Its C-terminal codes for a DNA-activable E3 SUMO ligase and promotes genome stability by targeting various proteins involved in DNA repair and fork progression [15, 17–20]. In mammals, NSE2 has a crucial role in cancer and aging suppression [21]. The presence of ubiquitin and SUMO ligase activities indicates that the Smc5/6 complex not only promotes genome integrity by organizing chromosomes, but also by signaling and regulating other cellular activities [22].

The yeast Smc5/6 complex promotes the removal of DNA junctions during DNA replication, facilitating sister chromatid disjunction [23–27]. Some of these connections are recombination intermediates, and Smc5/6 mutants are rescued, to different extents, by inactivation of recombination-promoting activities in yeast [23, 28]. In addition, Smc5/6 mutants show altered superhelical tension at forks [29, 30] and accumulate replication intermediates, which may also persist until anaphase and further prevent chromosome disjunction [23, 25, 31]. One of the main contributors to unwarranted junctions in Smc5/6 yeast mutants is Mph1 [32], the homologue of the FANCM motor protein [33]. In vitro, Smc5 directly inhibits the ability of Mph1 to anneal nascent strands on model replication forks, an activity known as fork reversal, which leads to the conversion of a three-way junction (similar to a replication fork) into a four-way junction resembling a Holliday junction [34]. Thus, Smc5/6-dependent restriction of Mph1 activity effectively reduces the X-shaped junctions at forks. Whether Smc5/6 directly regulates the human FANCM protein is currently unknown.

In human cells, replication forks are dynamically remodeled to safeguard the genome, particularly under conditions of replicative stress, through processes that involve nascent strand unwinding, reannealing, nucleolytic processing or repriming. One of the most common fork protective mechanisms involves the reversal of the fork in a process catalyzed by DNA translocases and RAD51 [35–37]. Because of reversal activities, the progression of forks is temporarily arrested in response to replicative stress, leading to a global reduction in fork speed, and helping to protect forks until conditions improve for restart. The mammalian Smc5/6 complex has also been linked to fork progression and processing. A recent study indicates that Smc5/6 contributes to the recruitment of fork protection factors, including FANCD2 and FANCM, to stalled replication forks under conditions of replicative stress [38]. In addition, depletion of the Smc5/6 complex in mammalian cells disrupts S-phase, leading to chromosome segregation errors [26, 39, 40].

The RING domain in the NSE1 protein is characterized by the presence of highly conserved cysteine and histidine residues which collectively coordinate two zinc atoms in a cross-brace conformation [41]. Proper folding of the RING domain is required for normal cell growth and DNA damage repair in yeast [41, 42]. Despite being evolutionarily conserved, its relevance and function in human cells remain unknown. To fill this gap, we used CRISPR-Cas9 to create stable cell lines with mutations in the human *NSMCE1* gene (hereafter referred to as *NSE1*). Our analysis shows that the RING domain is essential for NSE1 stability in HEK293T cells. Point mutations in essential zinc-coordinating residues or truncations of this domain lead to extremely reduced NSE1 protein levels and concomitant depletion of other Smc5/6 subunits. Despite *nse1* mutant cells being viable, they show extreme growth reduction and increased genomic instability, which we propose to stem from impairment of fork progression. Importantly, we demonstrate that Smc5/6 and FANCM exhibit synthetic sickness, and that the roles of Smc5/6 at replication forks and in maintaining genome integrity are independent of FANCM function. This indicates that the Smc5/6-dependent regulation of this fork reversal enzyme is not evolutionarily conserved.

## Results

### Mutation of the NSE1 RING domain in human cells

**Fig. 1:**
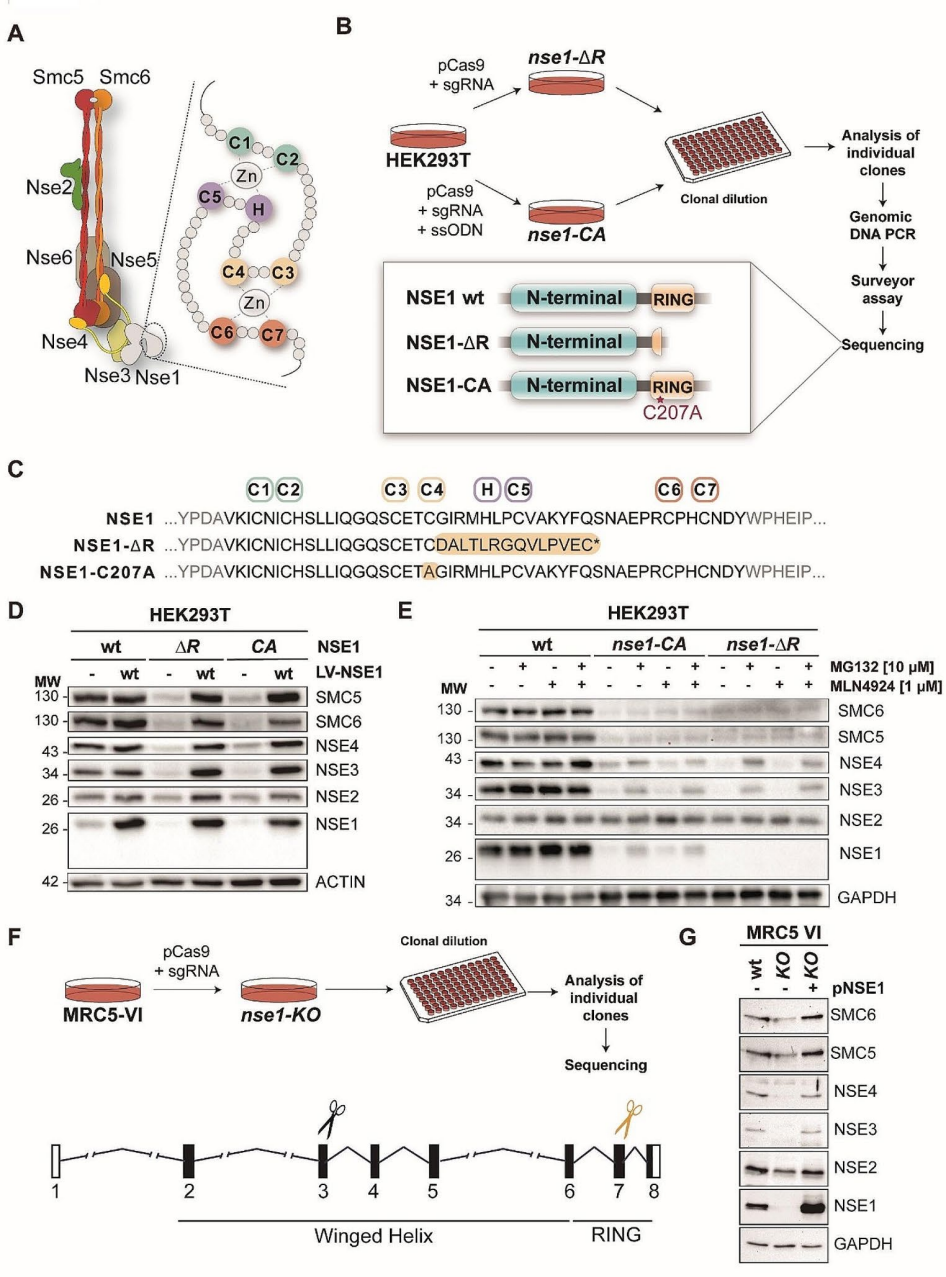
Mutation of the *NSE1* RING domain in human cells. **A**. Scheme of the NSE1 RING domain and location within the Smc5/6 complex. Residues necessary for zinc coordination are highlighted. **B**. CRISPR-Cas9 was used to generate either deletions or point mutations on the RING domain of human NSE1. **C**. Alignment of the wild-type C-terminal NSE1 sequence (RING domain is labeled in black) and two different individual clones identified. Conserved residues required for the coordination of Zinc atoms are depicted. Point mutation on C4 (C207A) position and RING truncation (ΔR) are labelled in yellow. **D**. Western blot analysis of NSE1 and the indicated subunits of the Smc5/6 complex. NSE1 protein could not be detected in the *nse1-*Δ*R* or *nse1-CA* mutants. Mutant cell lines also exhibited much lower levels of other Smc5/6 subunits. Complex levels could be rescued by the ectopic expression of wild type (wt) NSE1 from lentiviral vectors (LV-NSE1). **E**. Western blot analysis of cells expressing wild type or *nse1-CA*, or *nse1-∆R* mutant proteins, treated or not with the proteasome inhibitor MG132 (10 µM for 24 h) or/and the neddylation inhibitor MLN4924 (1 µM for 24 h), showing that the *nse1-CA* mutant protein is targeted for proteasomal degradation. No stabilization of the *nse1-∆R* mutant version was observed. **F**. Workflow for CRISPR to generate *nse1-KO* MRC5-VI cells. Black scissors mark position in exon 3 where Cas9 induces a DSB, within the winged helix domain coding sequence; for comparison, yellow scissors indicate the position in exon 7 used for CRISPR in HEK293T cells. **G**. Western blot analysis of NSE1 and the indicated subunits of the Smc5/6 complex in wt and *nse1-KO* (KO) MRC5-VI cells, transfected (+) or not (-) with a plasmid (pNSE1) expressing wild type NSE1.

The RING domain in NSE1 has a C_4_HC_3_ arrangement of zinc-coordinating residues, with the first and third pairs of residues (C1:C2 and H:C5) coordinating one zinc atom, and the second and fourth pairs (C3:C4 and C6:C7) coordinating a second zinc atom (Fig. 1A) [8]. Based on this structure, we generated two different *NSE1* mutants by CRISPR/Cas9: (i) a frame-shift mutation next to C207, corresponding to the fourth zinc coordination residue (C4) in the RING domain, to truncate the RING and C-terminal end of the protein (hereafter called *the nse1-ΔR* mutant); and (ii) a single point C4 mutation (hereafter called *nse1-CA*) (Fig. 1B). In the first case, we transfected HEK293T cells with a plasmid that carried a guide RNA (sgRNA) against exon seven, Cas9 endonuclease and GFP, which was used as a fluorescent marker to isolate individual transfected cells (Fig. 1B). Cas9-induced double-strand breaks (DSBs) can be repaired by non-homologous end-joining (NHEJ), leading to insertions or deletions (INDELs) next to the C207 codon. To introduce a C207A point mutation, we used a single-stranded oligodeoxynucleotide (ssODN) carrying the C207A mutation as a homologous DNA donor to promote homology-directed repair [43]. After clonal dilution and expansion, we evaluated the presence of mutations using PCR and the SURVEYOR assay. Clones with point mutations were identified by PCR and restriction analysis and analyzed by DNA sequencing to select homozygotic *nse1-ΔR* and *nse1-CA* mutants (Fig. 1B). The *nse1-ΔR* clone carried a deletion of 8 bp in exon 7, leading to a shorter protein carrying 14 missense residues immediately after C207 (Fig. 1C).

### NSE1 RING mutants destabilize the Smc5/6 complex

Next, we analyzed the expression of mutant proteins by western blotting. Due to the loss of the C-terminal residues in the *nse1-ΔR* mutant, we predicted a smaller protein, from 30.9 KDa in the wild type to 25.4 KDa in the *nse1-ΔR* mutant. Surprisingly, we could not detect the NSE1 protein in truncation mutants and only a faint band in point mutants (Fig. 1D). The protein levels of other subunits of the Smc5/6 complex were also extremely low in the *nse1-ΔR* or *nse1-CA* mutants, suggesting that their stability was affected by mutations in the RING domain of *NSE1* (Fig. 1D). Differently to most subunits of the complex, the NSE2 protein levels were not affected by mutations in *NSE1*. This is consistent with previous studies of siRNA knock down that demonstrate that all subunits, except NSE2, are required for the stability of the Smc5/6 complex [44]. The expression of wild-type NSE1 in *nse1-ΔR* or *nse1-CA* mutant cells using a lentiviral vector rescued Smc5/6 protein levels (Fig. 1D). In addition, the integrity of the Smc5/6 complex in *nse1-ΔR* cells was rescued to wild type conditions upon expression of NSE1, as assessed by immunoprecipitation of SMC5 (Supplementary Fig. 1A). These results suggest that *nse1* RING mutants are highly unstable, leading to concomitant instability of the Smc5/6 complex because of a missing NSE1 subunit or incorporation of a misfolded NSE1 protein.

To test whether the NSE1 mutant proteins and other Smc5/6 subunits are targeted for proteasomal degradation, we treated cell cultures with inhibitors of the ubiquitin-proteasome system. In particular, the expression of the NSE1-CA mutant protein increased to levels detectable by Western blotting after treatment of cells with the proteasome inhibitor MG132 (Fig. 1E), suggesting that mutation of C4 targets the protein for ubiquitination and degradation. Inhibition of CRL/SCF E3 ubiquitin ligases with MLN4924 had a weaker effect on NSE1-CA protein levels than MG132, pointing to a potential role of these ligases in targeting RING mutants for proteasomal degradation. In contrast, inhibition of other degradation pathways like autophagy or lysosomal degradation had no effect on the expression of NSE1-CA or other Smc5/6 subunits (Supplementary Fig. 1B). Differently to NSE1-CA, the NSE1-ΔR mutant protein was not detected after treatment with MG132 or MLN4924 (Fig. 1E), indicating that it cannot be stabilized by inhibition of the proteasome, at least under our experimental conditions. These results indicate that the integrity of the RING domain of NSE1 is required for the stability of the NSE1 protein and the Smc5/6 complex.

Despite the low expression of Smc5/6 subunits, *nse1* mutant cells were viable. While the Smc5/6 complex has proven essential for growth across various model organisms, from yeast to mammals, our findings suggest that NSE1 and the Smc5/6 complex might be dispensable for proliferation in specific human cell lines. To confirm this possibility using a completely different setup, we used CRISPR-Cas9 in MRC5-VI cells, a transformed lung fibroblast cell line, to target exon 3 of *NSE1* and truncate the protein near its N-terminus (Fig. 1F). This resulted in viable *NSE1* knockout cells (*nse1-KO*). Expression of the NSE1 protein was not detected in *nse1-KO* cells (Fig. 1G). Moreover, expression of NSE3 and NSE4 were almost undetectable, while expression of SMC5, SMC6 and NSE2 were significantly reduced, relative to wild type cells. Notably, the reintroduction of a wild-type copy of NSE1 restored the expression of all Smc5/6 subunits (Fig. 1G). Overall, we conclude that in the absence of NSE1, the human Smc5/6 complex becomes unstable.

### Differential contribution of the two zinc-coordinating centers in Smc5/6 protein levels

**Fig. 2:**
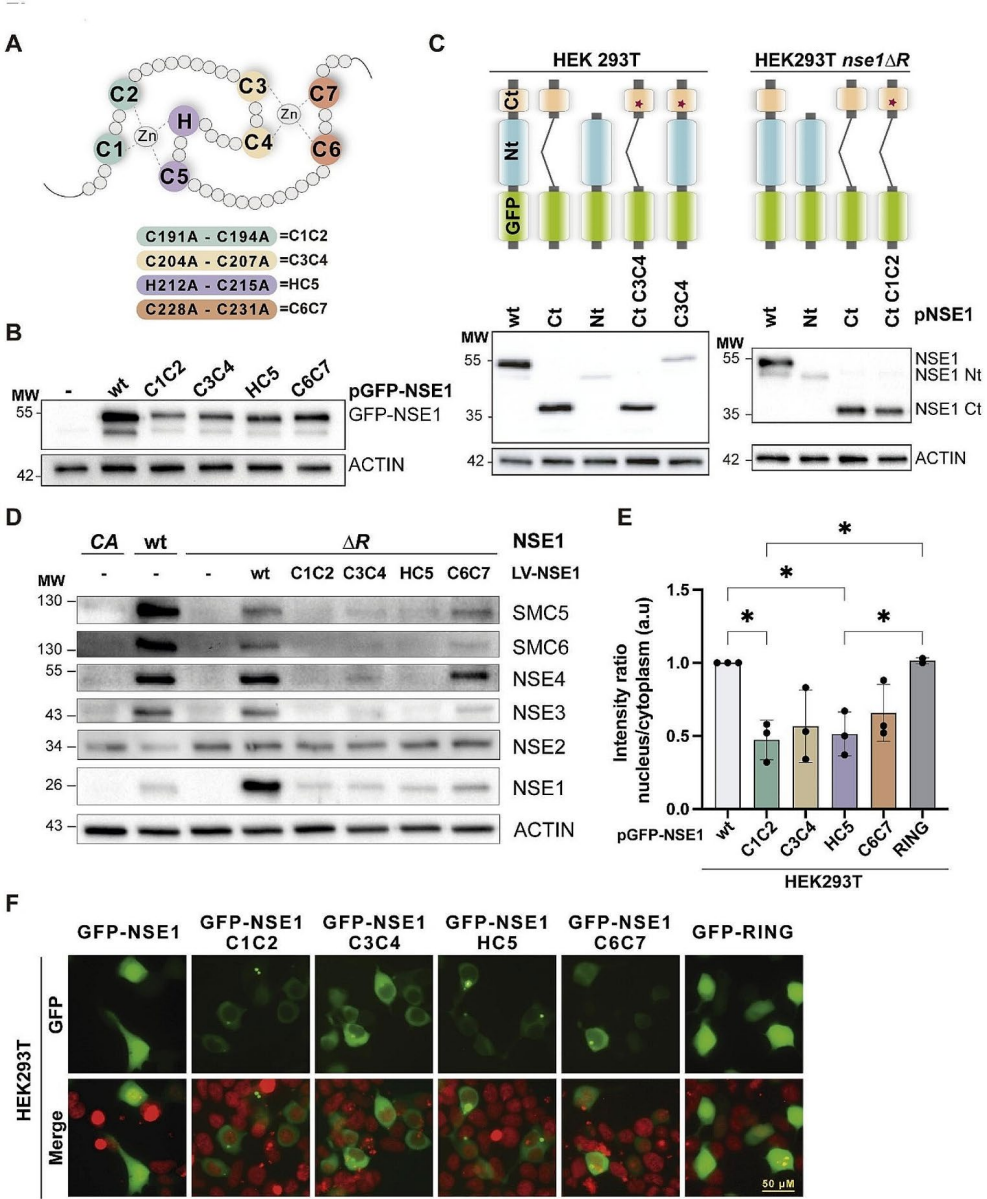
Differential contribution of the two zinc-coordinating centers in Smc5/6 protein levels. **A**. Scheme of the NSE1 RING domain and mutations in pairs of zinc-coordinating residues used in this study. **B**. Western blot analysis of HEK293T cells transfected with GFP-NSE1 fusions of wild type or the indicated RING mutants. **C**. Western blot analysis of cells expressing wild type or mutant GFP-NSE1 fusions. Ct = C-terminal RING domain of NSE1; Nt = N-terminal domain, excluding the RING. RING mutations are indicated with a star symbol. **D**. Western blot analysis of HEK293T *nse1-ΔR* cells infected with the indicated NSE1-expressing lentiviral vectors. **E.** Quantification of in vivo fluorescence microscopy of cells expressing GFP fusions of NSE1, either wt or Cys to Ala point mutants. Means and SD values of three experiments are shown. *, *P* < 0.05 by a one-way ANOVA followed by Tukey’s multiple comparison test. **F**. Representative microscopy images of cells counted in E. GFP is shown in green, nuclei in red. Note the presence of nuclear exclusion in RING mutants, which is not observed in wild type NSE1 (left panel) or fusions of GFP to the RING domain (right panel)

The C4 mutation targets the second zinc-coordinating center in the NSE1 RING domain (Fig. 2A). To dissect the contribution of each zinc-coordinating domain in NSE1 function, we introduced double point mutations in NSE1 expression vectors to target pairs of zinc-coordinating residues: C191A-C194A (C1C2), C204A-C207A (C3C4), H212A-C215A (HC5) and C228A-C231A (C6C7) (Fig. 2A). The *NSE1* RING mutant alleles, tagged N-terminally with GFP, were transfected into HEK293T cells. The four RING mutant proteins were expressed at significantly lower levels than wild-type NSE1, suggesting that they are more unstable, with C1C2 being the most and C6C7 the least affected (Fig. 2B). These results suggest that unfolded RING mutant domains might be recognized by protein quality control pathways and targeted for destruction. To test this, we created truncated versions of the GFP-NSE1 protein that only express the N- or C-terminus (Fig. 2C). The NSE1 protein lacking the RING domain (Nt-NSE1) exhibited substantially lower expression compared to the full-length NSE1 or the C-terminal domain (Ct-NSE1) (Fig. 2C). The decreased expression of Nt-NSE1 could be rescued by inhibition of the proteasome, confirming that the absence of the RING domain renders NSE1 unstable (Supplementary Fig. 2). Surprisingly, the introduction of C3C4 mutations had negligible effects on Ct-NSE1 expression levels (Fig. 2C), indicating that an unfolded RING domain does not inherently lead to instability and is unlikely to be directly recognized for destruction. Similar results were obtained when the Nt-NSE1, Ct-NSE1 or Ct-NSE1 C1C2 mutants were expressed in *nse1-ΔR* mutant cells (Fig. 2C).

Next, we evaluated the ability of the RING mutant to rescue Smc5/6 protein levels in *nse1-ΔR* mutant cells. As shown in Fig. 2D, C1C2 and HC5 mutants in the RING domain did not recover expression of Smc5/6 subunits. Only C6C7 and, to a lesser extent C3C4, were able to slightly rescue Smc5/6 protein levels (Fig. 2D). These results suggest that the first set of zinc-coordinating residues in the NSE1 RING domain (C1, C2, H, and C5) is more relevant for the overall function of the NSE1 RING domain in Smc5/6 complex stability than the second set (C3, C4, C6, and C7).

Of note, wild-type GFP-NSE1 was localized in both the nucleus and the cytoplasm, while RING mutants showed partial nuclear depletion (Fig. 2E and F). Quantification of microscopic images showed that GFP-NSE1 nuclear localization was diminished in all RING mutants, although mean values were only statistically significant for mutations in the first zinc-coordination center (Fig. 2E). This observation suggests that the RING domain in NSE1 contributes not only to the stability of the Smc5/6 complex but also to promote its nuclear localization. Alternatively, RING mutant proteins might be targeted for destruction when localized to the nucleus. Proteasome inhibition with MG132 increased the nuclear localization of C3C4- and Nt-NSE1 mutants (Supplementary Fig. 3), suggesting that RING mutants might be targeted for degradation in the nucleus. Overall, we conclude that NSE1 and other subunits of the Smc5/6 complex become unstable in the absence of a folded RING domain in human cells, with the first zinc-coordination center playing a more relevant role in Smc5/6 complex stability and localization than the second one.

### NSE1 RING mutant cells exhibit reduced proliferation and increased genomic instability

**Fig. 3:**
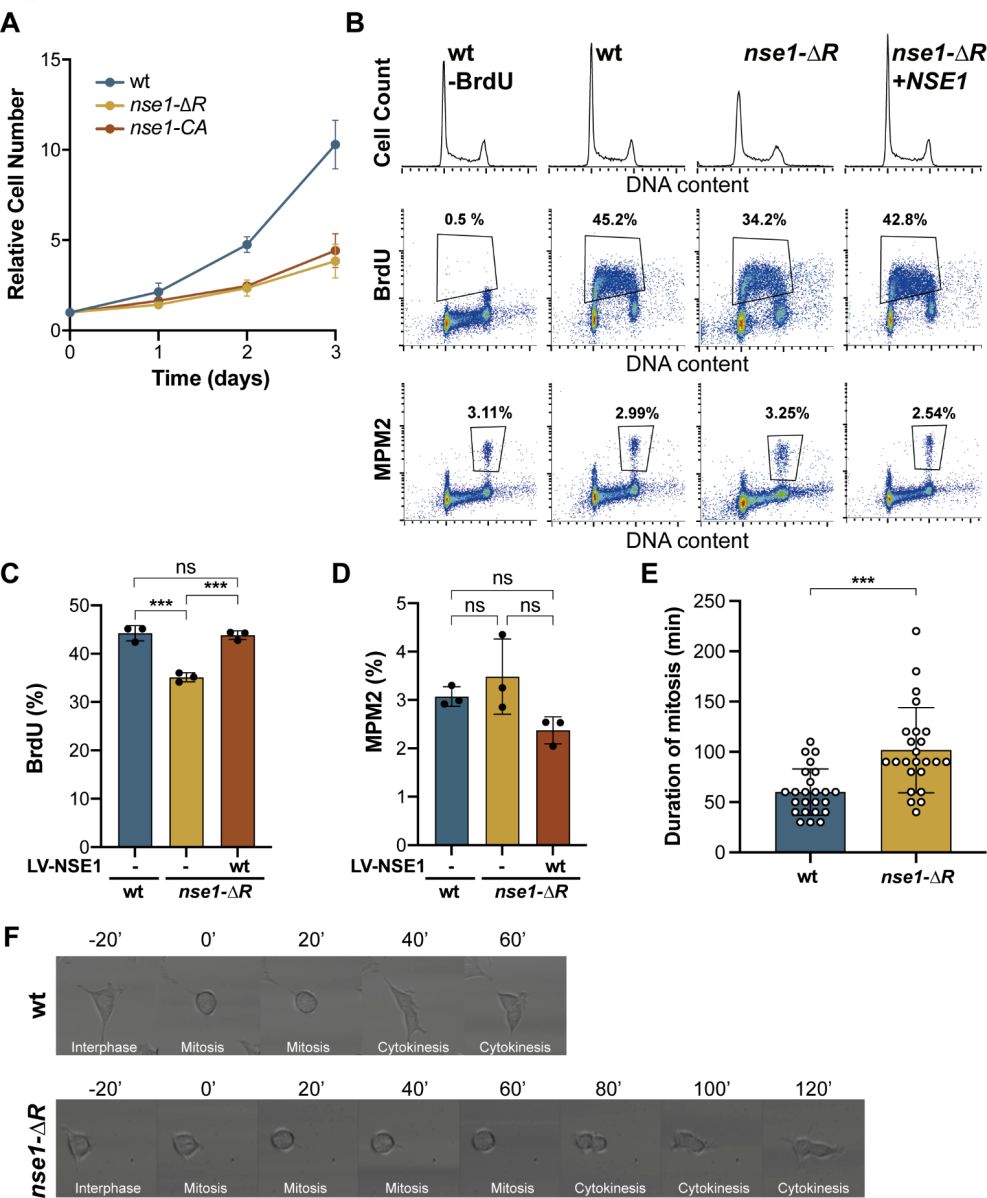
Mutations in the RING domain of NSE1 cause growth and cell cycle defects. **A**. Growth curve analysis of wild type cells and two individual *nse1* mutants. Cell proliferation was followed for 3 days using Trypan blue (TB) exclusion assay. Mean and SEM values from three independent experiments are shown. **B**. DNA content, cells in S-phase (BrdU positive) and cells entering Mitosis (MPM2 positive) in wt, *nse1-∆R* mutants, and mutant cells ectopically expressing wt NSE1, were analyzed by flow cytometry. Values are representative of three replicas. **C**. Quantification of BrdU positive cells from FACS, showing mean and SD. ***, *P* < 0.001 by a one-way ANOVA followed by Tukey’s multiple comparison test. **D**. Quantification of MPM2 positive cells from FACS, showing mean and SD. ns, not significant. **E**. Quantification of the duration of mitosis in wild type and *nse1-ΔR* mutants from time-lapse microscopy. Mitotic progression was followed for 48 h and images were taken every 10 min. Bars represent mean mitotic time. Dots are the duration of mitosis for individual cells, error bars are SD values. *N* = 24 for each cell line. ***, *P* < 0.001 by a Student’s t-test. **F**. Representative time-lapse images showing the mitotic progression of wild type cells and *nse1-ΔR* mutants

Interestingly, all *nse1* mutants analyzed in this study showed a slower proliferation rate (Fig. 3A and Supplementary Fig. 4A). To better understand whether *nse1* mutants are delayed at specific cell cycle stages, we studied the cell cycle distribution by flow cytometry. *nse1-ΔR* mutant cultures showed smaller and broader peaks of G1 and G2/M than wild-type cells (Fig. 3B), probably reflecting a greater variability in chromosome copy number, with little alteration in the relative proportion of G1 and G2/M cells. The *nse1-ΔR* culture showed a lower fraction of cells in S-phase, as determined by labelling with BrdU (Fig. 3B and C). However, there was no significant difference in the proportion of cells undergoing mitosis, as indicated by MPM2 positivity, between the wild-type and *nse1-ΔR* cultures. (Fig. 3B and D).

To further analyze the length of M phase, we used time-lapse microscopy. HEK293T cells are characterized by an epithelial cell morphology but become rounded when entering mitosis. We thus measured the time spent by wild type and *nse1-ΔR* cells in the round-shaped morphology, before undergoing cytokinesis. *nse1-ΔR* cells took longer time to complete mitosis than wild-type cells and displayed, on average, a one-hour increase in the duration of M phase (Fig. 3E and F). Growth defects, alterations in the cell cycle profile, and BrdU incorporation of RING mutants could be rescued by ectopic expression of wild-type NSE1 (Fig. 3B-D and Supplementary Fig. 4), indicating that (i) the observed phenotypes are specifically due to mutation of the RING domain and (ii) the unstable NSE1 mutant protein does not exert a dominant negative effect. Overall, these results indicate that the S and M phases were differentially affected in *nse1* mutant cells.

**Fig. 4:**
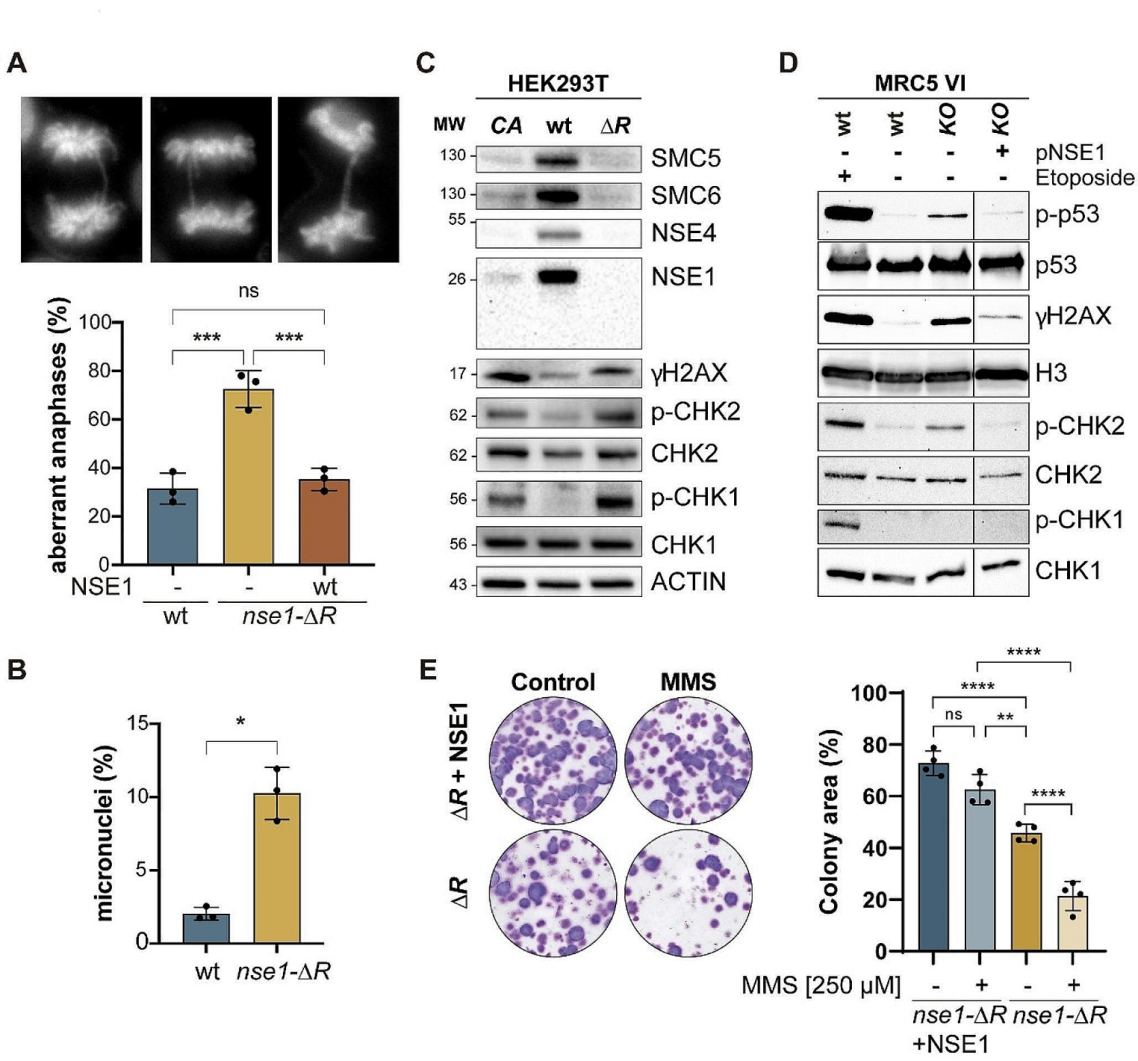
*nse1* mutants show increased levels of DNA-damage and genomic instability. **A.** Up, representative images of aberrant anaphases; bottom, frequency of aberrant anaphases (anaphase bridges and lagging chromosomes), relative to the total number of anaphases scored; a minimum of 80 anaphases were counted per experiment; bars indicate means and error bars SD. **B.** Frequency of micronuclei; bars indicate means and error bars SD. **C**. Western blot analysis of the indicated Smc5/6 subunits and different markers of endogenous DNA damage (γH2AX) and checkpoint activation (phospho (S345) CHK1, phospho (T48) CHK2) in HEK293T cells. **D**. Western blot analysis of DNA damage checkpoint activation markers in wild type and *nse1-KO* MRC5-VI cells. Where indicated, cells were treated with etoposide 5 µM for 16 h to induce DNA damage. **E**. Left: Clonogenic assay of HEK293T *nse1-ΔR* cells expressing (+) or not (-) *NSE1* after acute exposure (2 h) to 250 µM MMS. Right: quantification of colony intensity from three independent experiments. Dots are individual clonogenic assay measures. Error bars are SD values. In A, B and E: ns, not significant; *, *P* < 0.05; **, *P* < 0.01; ***, *P* < 0.001; ****, *P* < 0.0001 by a one-way ANOVA followed by Tukey’s multiple comparison test

Careful inspection of the flow cytometry profiles further revealed that *nse1* mutants had more cells with DNA content below G1 or above G2/M (Supplementary Fig. 4B), suggesting defects in genomic stability. We observed a similar effect of the *nse1-KO* mutation in MRC5-VI cells, with an accumulation of cells with more than 4 N DNA content (Supplementary Fig. 4C, D). This could be due to higher levels of endogenous DNA damage, as previously described for other Smc5/6 mutants, in accordance with the role of the Smc5/6 complex in chromosome segregation and in maintaining the stability of the genome [26, 40]. Quantification of anaphase cells with lagging chromosomes or chromatin bridges showed that *nse1-ΔR* substantially increased the number of aberrant anaphases, almost triplicating the incidence relative to wild-type cells (Fig. 4A). A similar effect was observed in *nse1-CA* cells (Supplementary Fig. 5A). Ectopic expression of wild type *NSE1* in *nse1-ΔR* cells rescued chromosome segregation defects to wild type levels (Fig. 4A). Chromosome segregation and DNA repair defects frequently result in the formation of micronuclei (MN), extranuclear bodies containing chromosomes (or chromosome fragments) that remain in the cytoplasm after cell division. As shown in Fig. 4B, *nse1-ΔR* mutants had higher levels of MN. To further assess the stability of the genome in *nse1* RING mutants, we analyzed various targets of DNA damage and/or replicative checkpoint, including phosphorylation of the CHK1 and CHK2 checkpoint effector kinases and histone H2AX. CHK1 is phosphorylated and activated in response to replicative stress, whereas CHK2 is primarily involved in the response to DSBs. As shown in Fig. 4C, the levels of phosphorylated histone H2AX (γH2AX) were higher in *nse1-ΔR* or *nse1-CA* mutants than in wild-type cells. Additionally, we detected higher levels of phosphorylated CHK1 and CHK2 (Fig. 4C). These results indicate that *nse1* RING mutant cells have problems during DNA replication and higher levels of chromosome breaks. Interestingly, *nse1-KO* cells behaved similarly in terms of H2AX and CHK2 phosphorylation but did not show CHK1 activation (Fig. 4D). This could be due to genetic differences between MRC5-VI and HEK293T cell lines, including a normal diploid karyotype in MRC5-VI cells versus a more complex karyotype in the latter, resulting in differential activation of the replicative checkpoint. To confirm that *NSE1* mutant cells exhibited genomic instability, we measured sister chromatid exchange (SCE) events, which result from the physical exchange of DNA between sister chromatids. Although SCEs occur naturally, they increase in response to higher levels of double-stranded breaks or replication fork-stalling events. The *nse1-ΔR* mutant cells showed significantly elevated endogenous levels of SCE compared to wild-type cells (Supplementary Fig. 5B).

Yeast *nse1* RING mutants are hypersensitive to genotoxic stress [41, 42]. To evaluate whether *nse1* RING mutant cells showed altered sensitivity to external DNA damage, we treated *nse1-ΔR* mutant cells and *nse1-ΔR* mutant cells ectopically expressing wild-type *NSE1* with the alkylating agent MMS. As shown in Fig. 4E, *nse1-ΔR* mutant cells were more sensitive to MMS than *nse1-ΔR* cells rescued by *NSE1* expression. Rescue experiments with RING mutants indicated a differential contribution to MMS sensitivity of the two zinc-coordinating residues, with the first center (C1, C2, H and C5) being more relevant than the second one (C3, C4, C6 and C7; Supplementary Fig. 6), in accordance with their effects on Smc5/6 complex stability and NSE1 localization (Fig. 2). Our observations suggest that the higher levels of genomic instability sensitizes *nse1-RING* mutants to exogenous DNA damage.

### FANCM and Smc5/6 independently contribute to fork progression and genome integrity in human cells

**Fig. 5:**
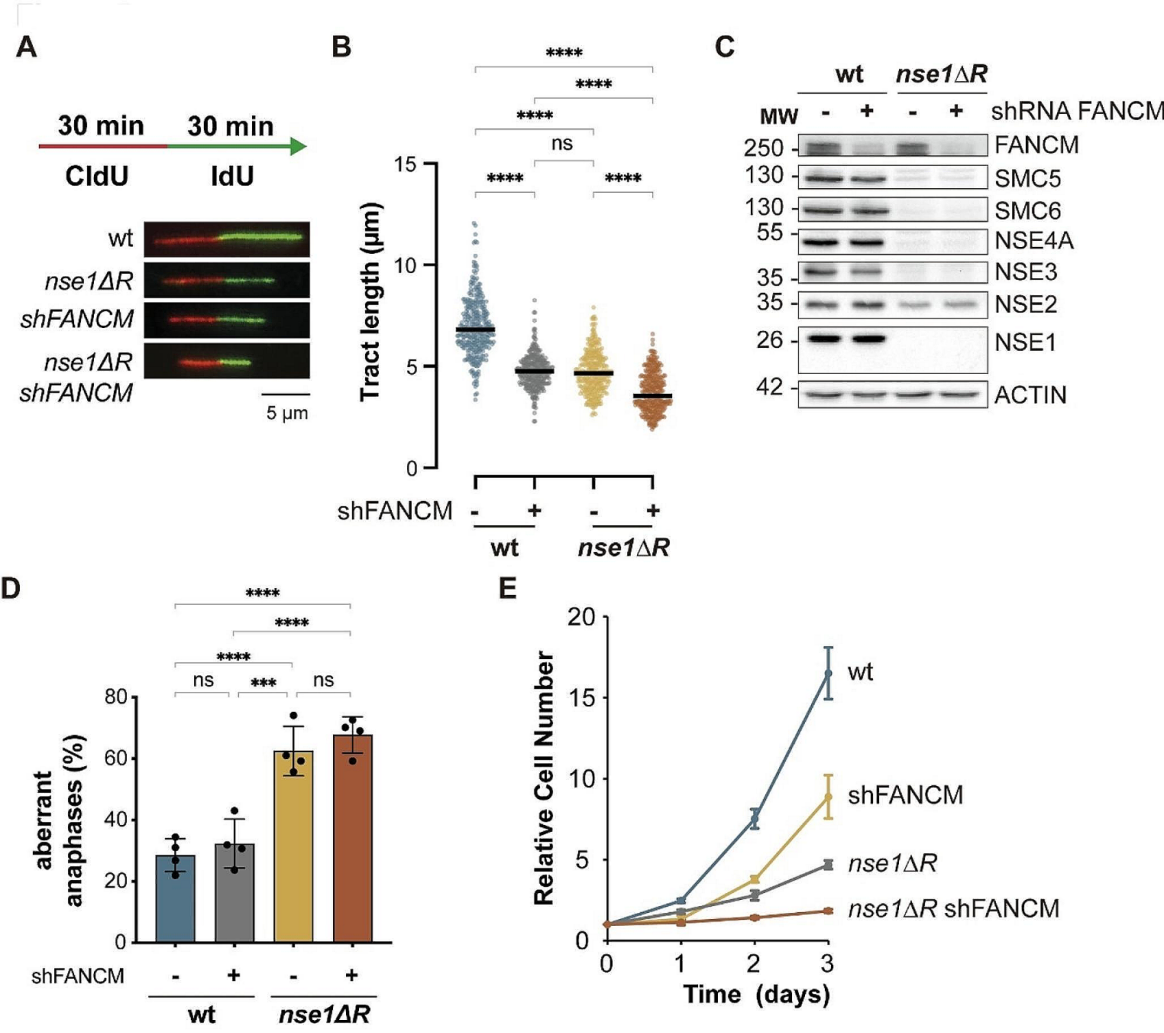
The Smc5/6 complex promotes replication fork progression in a FANCM-independent manner in human cells. **A**. Cells were pulse-labelled with CldU for 30 min and with IdU for an additional 30 min and subjected to DNA fibers analysis. Representative images of DNA fibers obtained from wild type and *nse1-ΔR* mutant cells, transduced with a scrambled shRNA (-) or an shRNA against FANCM (+) to down-regulate its expression. **B**. The length of the IdU tracks in DNA fibers from cells with the indicated genotype, measured by ImageJ Software. Representative experiment from three independent repetitions. ns, not significant; ****, *P* < 0.0001 by a Kruskal-Wallis followed by Dunn’s multiple comparisons test. C. Western blot analysis of FANCM and subunits of the Smc5/6 complex in cells of the indicated genotype (wt and *nse1-ΔR*) transduced with a mock shRNA (-) or with an shRNA to downregulate FANCM (+). **D**. *nse1-ΔR* cells accumulate a higher frequency of aberrant anaphases. Mean and SD values of three experiments are shown, with not significant (ns) differences upon down-regulation of FANCM. ***, *P* < 0.001; ****, *P* < 0.0001 by a one-way ANOVA followed by Tukey’s multiple comparison test. **E**. Growth curve analysis of wt and *nse1-ΔR* HEK293T cells transduced with a scrambled shRNA or with shFANCM. Proliferation was followed for 4 days. Mean and SD values are shown

The activation of the S phase checkpoint suggests that *nse1-ΔR* cells might experience defects in replication fork progression. To analyze this possibility, we performed DNA fiber analysis in wild-type and mutant HEK293T cells. We first treated cultures for 30 min with the thymidine analogue CldU to label ongoing forks. Next, we labelled cells for 30 min with a second analogue, IdU, and examined fork progression by DNA spreading, using the length of the second halogenated nucleotide as a proxy for fork speed (Fig. 5A). As shown in Fig. 5B, the replication tracks in *nse1-ΔR* cells were shortened about 30%, relative to wild type cells, indicating that forks proceed at a slower speed in the absence of the Smc5/6 complex. We observed a similar reduction in fork speed in *nse1-CA* mutant cells (Supplementary Fig. 5C). Fork speed was also significantly reduced after rapid Smc5/6 inactivation in double auxin inducible degron (AID) SMC6-AID NSE4A-AID HCT116 cells (Supplementary Fig. 5E and F). Importantly, auxin-mediated depletion of SMC6 and NSE4A did not activate CHK1 under these experimental conditions (Supplementary Fig. 5D), suggesting that the altered fork rate in Smc5/6 mutants is not caused by previous problems in chromosome segregation or by activation of checkpoints.

The Smc5/6 complex has been shown to promote replication fork progression in the rDNA under normal conditions [24], and elsewhere in response to DNA damage [25] in budding yeast. These functions seem to be dependent on restriction of the fork reversal activity of the Mph1 motor protein, the yeast homologue of FANCM [32, 34]. To test if FANCM is involved in the reduction of replication fork rate, we transduced HEK293T cells with a vector expressing an shRNA to knock down FANCM expression. As shown in Fig. 5C, FANCM protein levels were substantially reduced in both wild type and *nse1-ΔR* cells. Downregulation of FANCM in wild type cells led to reduced fork progression in wild type cells, to levels very similar to *nse1-ΔR* cells (Fig. 5B). However, simultaneous downregulation of Smc5/6 and FANCM in *nse1-ΔR* cells led to further restrain in fiber length. This observation indicates that, differently to yeast, the replication defects in Smc5/6 mutants cannot be rescued by depletion of FANCM. In addition, it suggests that FANCM and Smc5/6 independently contribute to replication fork progression in human cells. The role of the Smc5/6 complex on chromosome replication is essential to promote the physical separation of sister chromatids in mitosis. To know if FANCM and Smc5/6 also independently contribute to later events in mitosis, we analyzed the presence of aberrant figures in anaphase cells. As shown in Fig. 5D, reducing FANCM expression marginally increased the occurrence of abnormal anaphase structures in both wild type and in *nse1-ΔR* cells. Although this effect was not statistically significant, it is worth noting that FANCM downregulation did not improve the growth of *nse1* mutant cells. In fact, FANCM depletion in *nse1-ΔR* mutant cells had a strong impact on cell proliferation, further revealing a synthetic sick phenotype in double *nse1 fancm* mutant cells (Fig. 5E). Altogether, these results suggest that, differently to yeast [34], FANCM is not restricted by the Smc5/6 complex in human cells.

**Fig. 6:**
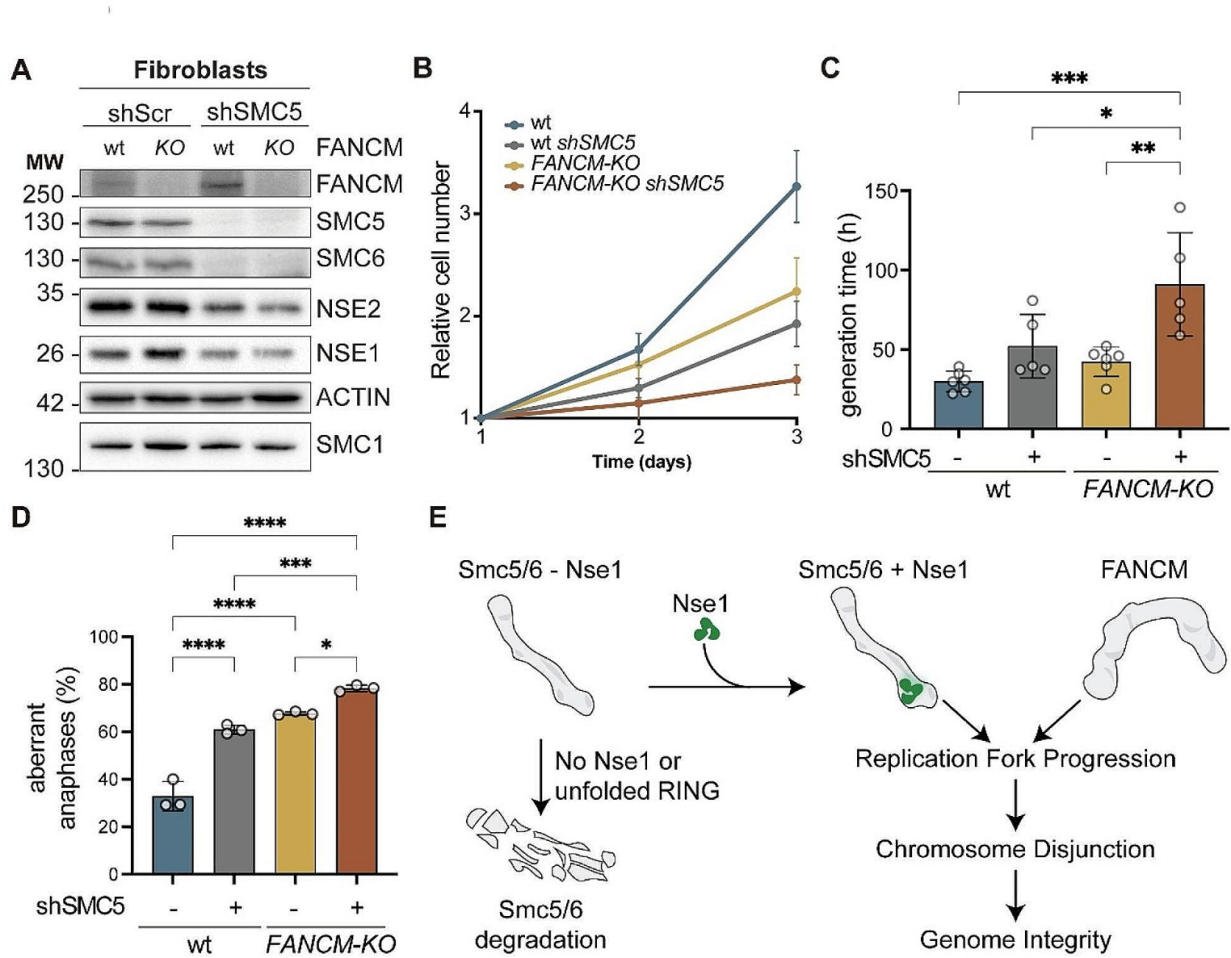
SMC5 and FANCM independently contribute to genome integrity in human fibroblasts. **A.** Western blot analysis of FANCM and Smc5/6 subunits in cells of the indicated genotype transduced with shRNA scramble or against *SMC5*. ACTIN and the SMC1 cohesin subunit were used as loading controls. **B.** Growth curve analysis. Proliferation was followed for 3 days using Trypan blue exclusion assay. Mean and SEM values are shown. **C.** Quantification of generation times from B. Circles indicate individual measurements from 5 independent experiments. Bars are mean values; error bars are SD. **D**. Quantification of aberrant anaphases in cells of the indicated genotype. Circles are individual measurements from 3 independent experiments; bars are mean values; error bars represent SD. **E.** Model for the role of the NSE1 RING domain in Smc5/6 and genome stability. Smc5/6 complexes assembled without NSE1 or carrying dysfunctional RING domains become rapidly degraded. Assembly of Smc5/6 subunits with NSE1 stabilizes functional complexes that work in parallel to FANCM to promote replication fork progression, completion of DNA replication and chromosome disjunction, contributing to the maintenance of genome integrity. In C and D: *, *P* < 0.05; **, *P* < 0.01; ***, *P* < 0.001; ****, *P* < 0.0001 by a one-way ANOVA followed by Tukey’s multiple comparison test

Since we could not conclude whether FANCM and Smc5/6 operate in the same pathway for chromosome disjunction, we tested a different model of FANCM inactivation, based on human fibroblasts derived from FANCM-KO patients. As a control for FANCM activity, we used the same FANCM-KO fibroblasts complemented with a wild type copy of FANCM under the control of a tetracycline-inducible promoter. Next, we inactivated Smc5/6 function by transducing cells with an shRNA to downregulate SMC5 expression. As shown in Fig. 6A, the *SMC5* shRNA reduced expression of the SMC5 and SMC6 subunits, with a more modest effect on NSE1 and NSE2 expression. This is most probably due to destabilization of other subunits of the complex upon loss of one of them. Knock down of *SMC5* in FANCM-wt fibroblasts reduced cell proliferation, increasing the generation time by almost 70% (Fig. 6B and C). FANCM-KO cells also grew more slowly, with a 30% increase in generation time relative to FANCM-wt cells. However, the simultaneous loss of FANCM and Smc5/6 downregulation had a synergistic impairment on growth, leading to a three-fold increase in the generation time relative to wild type cells (Fig. 6C). Moreover, FANCM-KO fibroblasts showed a clear defect in chromosome segregation, doubling the number of aberrant anaphases observed in wild type cells. Importantly, this effect was significantly worsened by knock down of *SMC5* in FANCM-KO fibroblasts (Fig. 6D), indicating that FANCM and Smc5/6 operate through different pathways to promote chromosome segregation. Overall, we conclude that Smc5/6 and FANCM independently contribute to replication fork progression and chromosome disjunction in human cells (Fig. 6E).

## Discussion

Here we have generated mutants in the *NSE1* gene in human cells and identified a critical role for the RING domain in the stability of the human Smc5/6 complex, with differential contribution of the two zinc-coordinating centers in the RING. In addition, we have shown that Smc5/6 depletion in *nse1* RING mutants interferes with replication fork progression and negatively affects genome integrity in a FANCM-independent manner, suggesting that the Smc5/6 complex promotes the progression of forks and the stability of the genome through pathways that are not necessarily conserved in evolution.

### The RING domain in the NSE1 protein is required for the stability of Smc5/6 subunits in human cells

The Smc5/6 complex is the only known SMC member with two RING-domain subunits, NSE2 and NSE1, each of them able to modify other proteins/complexes through SUMO and ubiquitin respectively [22]. We have recently characterized different elements, many of them related to ribosome biogenesis, as Nse1-dependent ubiquitin targets [10]. In addition to this signaling role, the RING domain in NSE1 may also coordinate protein-protein interactions within the Nse1-Nse3-Nse4 subcomplex in yeast [41, 42]. While we have not analyzed the effect of NSE1 mutations on ubiquitination, we speculate that the phenotypes described here for *nse1* mutants result mainly from loss of Smc5/6 stability and function.

The two mutants developed in this study are predicted to destroy RING interaction interfaces, either by truncation (*nse1-ΔR*) or by unfolding (*nse1-CA*) of the NSE1 RING domain. The expression of wild-type NSE1 effectively rescued all the tested phenotypes in *nse1* mutants, suggesting that they cannot be attributed to off-target effects generated by Cas9. Both mutants drastically reduced NSE1 protein levels, suggesting that RING-dependent interactions are necessary for NSE1 stability in human cells. Our results indicate that the NSE1-CA mutant protein is targeted for proteasomal destruction. By extension, we think that NSE1-ΔR may have a similar fate. Although we could not detect the endogenous NSE1-ΔR protein after proteasome inhibition, we observed stabilization of over-expressed C-terminal truncated versions after proteasome inhibition (Supplementary Fig. 2). In addition, RING mutations also reduce the expression of SMC5, SMC6, NSE3 and NSE4 subunits. The fact that these subunits can be partially stabilized by MG132 indicates that they are targeted for proteasome degradation, most probably through quality control pathways that recognize complexes with missing or aberrant subunits [45]. We speculate that the mutant NSE1 proteins might be more rapidly destroyed by the proteasome, leading to very low expression levels, even in cells treated with MG132. Our observation that NSE2 protein levels are relatively unaffected in *NSE1* mutant cells adds to previous studies suggesting that NSE2 may be regulated independently from other Smc5/6 subunits [30, 44, 46, 47]. However, other studies using conditional knock out models have also observed concomitant depletion of NSE2 upon SMC5 inactivation [48]. Expression of RING mutants is mostly affected when the N-terminal domain, which recruits NSE1 to the Smc5/6 complex, is present (Fig. 2), suggesting that RING mutants become destabilized after their attempted (and failed) assembly into Smc5/6 complexes (Fig. 6E). This suggests that the RING domain helps to hinder a degron motif, which can potentially target the Smc5/6 complex for degradation when exposed. The putative location of this degron is currently unknown. Our results also suggest that targeting for degradation may preferentially occur in the nucleus. It would be very interesting to know at which stage *nse1-RING* mutants become unstable. We hypothesize that it could require: (i) formation of an NSE1-NSE3-NSE4 subcomplex, (ii) integration into the Smc5/6 complex or (iii) dynamic association with DNA. Controlled degradation might serve as a quality control mechanism, ensuring the presence of only properly folded and active Smc5/6 complexes. Previous studies with the yeast subunits have already pointed to a role for the RING domain in the assembly of the Nse1-Nse3-Nse4 subcomplex in vitro [41], and this might be the principal reason for the Smc5/6 instability in *nse1-RING* mutants. The middle part of the Nse4 protein is threaded through a small cavity in the Nse1 and Nse3 subunits [49] and mutations affecting the NSE3-NSE4 interface have been shown to affect the stability of the human Smc5/6 complex, resulting in severe LICS syndrome [50]. Thus, it is possible that the RING domain participates in opening of NSE1 and/or NSE3 to promote the binding to the NSE4 subunit.

**Fig. 7:**
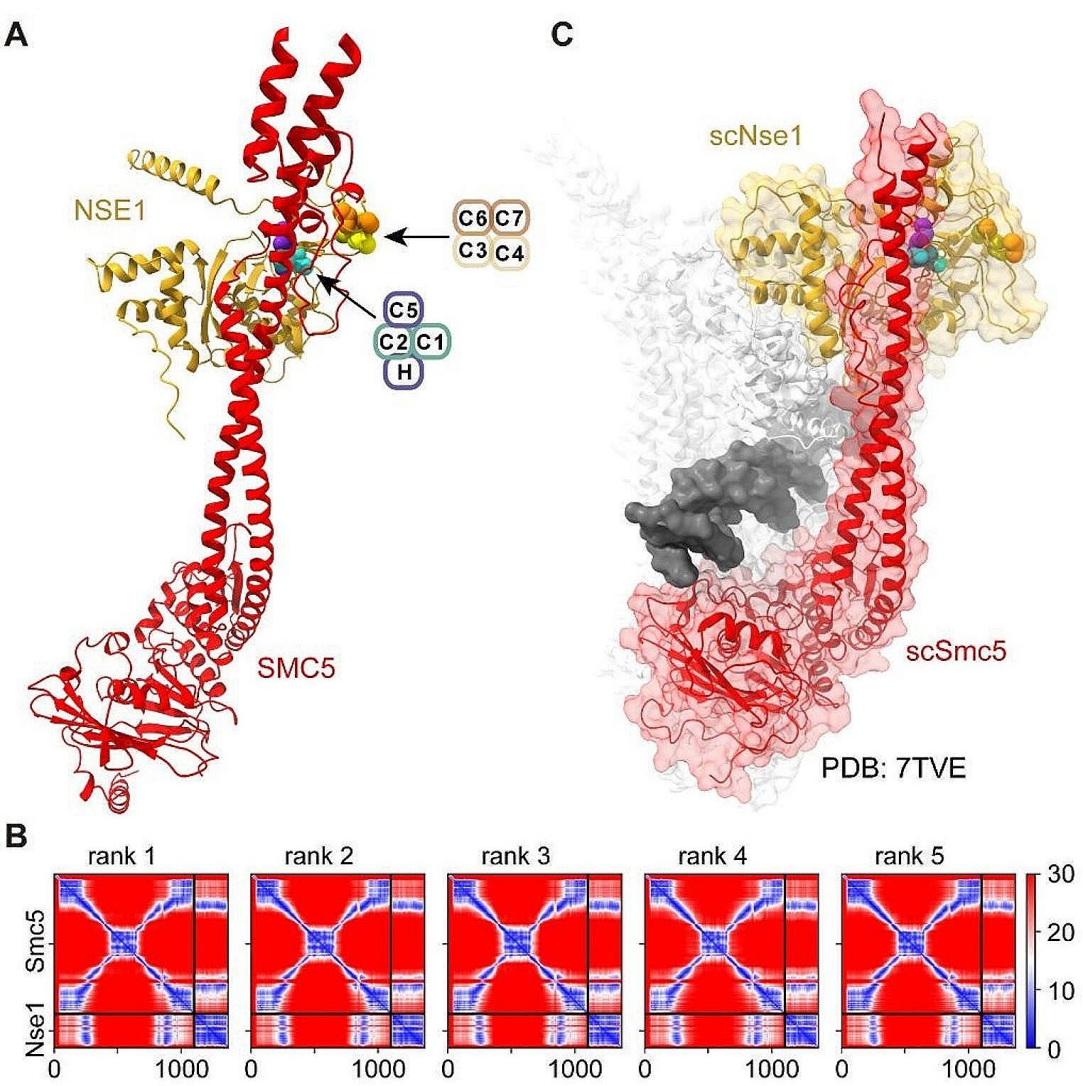
AlphaFold Multimer predicts close association of the first zinc-coordinating center in the NSE1 RING to the joint region in SMC5. **A**. Cartoon backbone representations of the AlphaFold Multimer predicted interaction between SMC5 and NSE1. Zinc-coordinating residues are shown as space-filled and colored as in Fig. 1 (turquoise for C1-C2, violet for H-C5, yellow for C3-C4 and orange for C7-C8). Note the closer association of the first zinc-coordinating center (C1-C2-H-C5) with the joint domain of SMC5, in comparison to the second zinc-coordinating center. **B**. Predicted Aligned Error (PAE) for the top-ranked SMC5-NSE1 AlphaFold Multimer models. **C**. Cryo-EM structure (PDB: 7TVE) of the budding yeast Smc5/6 complex in its DNA-bound form. Note the similar configuration of the SMC5-NSE1 interaction in the human model (A) and in the yeast structure (C)

On the other hand, the RING may have further roles in assembled and active Smc5/6 complexes, and it is also conceivable that the RING domain in NSE1 participates in direct protein-protein interactions with specific subunits within the Smc5/6 complex. In fact, recent structures of the yeast Smc5/6 complex have revealed the spatial rearrangement of the NSE subunits after engagement of the ATPase heads. In the absence of ATP, the RING domain projects out of the complex and is not involved in protein-protein interactions [4]. In the presence of ATP, the Nse1-Nse3 heterodimer relocates to the upper part of the ATPase heads; in this configuration, the RING domain in Nse1 binds the joint region of Smc5, with the first zinc-coordination center in contact with Smc5 [51]. Although this interaction is most probably transient in nature, occurring between head engagement and ATP hydrolysis, it might be functionally relevant. Two recent studies have analyzed interactions within the human Smc5/6 complex by crosslink and mass spectrometry [52, 53]. Even though they did not report interactions between NSE1 and SMC5, neither study analyzed the Smc5/6 complex when clamped on DNA. Remarkably, Alphafold Multimer predicted an interaction between the head proximal region of the SMC5 coiled domain, known as joint, and different regions in NSE1, including the RING domain (Fig. 7A and B). In this model, the first zinc-coordinating center directly contacted SMC5, with the possible participation of C2 (Cys194) in hydrogen bonding, while the second center was positioned at a greater distance from SMC5 (Fig. 7A). This configuration is similar to the cryo-EM structure of the budding yeast Smc5/6 complex in its DNA-bound form (Fig. 7C) [39]. Thus, in addition to the RING requirement for assembly of the NSE1-3-4 subcomplex, our model suggests that the more pernicious effects of mutations in the first zinc-coordination center could stem from an important role in stabilizing the DNA-bound state of the Smc5/6 complex. It is possible that the inability to reach this configuration might promote the destruction of Smc5/6 complexes while attempting to bind DNA, leading to Smc5/6 depletion in NSE1 RING mutants. At this stage, it is tempting to speculate that targeted degradation of wild type Smc5/6 molecules under specific configurations could also have a physiological role in chromosome organization.

### Disruption of the RING domain in NSE1 impairs genomic stability in human cells

*nse1* mutant cells progressed more slowly through mitosis and partially activated DNA damage responses. The lower proliferation rate may be partly due to diminished cell viability in the absence of Smc5/6 function. In parallel to lower viability of cell progeny, we think that all cell cycle phases may be extended similarly since we could not detect major alterations in the G1 or G2 FACS profile. *nse1* mutant cultures also accumulated cells with altered DNA content, most probably reflecting defects in chromosome segregation. In accordance, *nse1* mutant cells showed higher levels of micronuclei and aberrant anaphases. We think that depletion of NSE1 promotes the formation of pathological structures during chromosome replication. This would result in the accumulation of anaphase bridges, broken chromosomes and higher levels of genomic instability and DNA damage sensitivity. Importantly, the genomic instability defects of *nse1* mutant cells are reversible, as they could be rescued by re-expression of wild type NSE1. Interestingly, a recent study has shown that acute depletion of Smc5/6 subunits in G1-arrested cells does not alter S phase or mitotic entry, despite leading to chromosome non-disjunction in the first anaphase [26], similarly to what has been described for *smc5/6* mutants in yeast [23–25]. In accordance, patient-derived cell lines with mutations in NSE3, SMC5, or SLF2 show increased aberrant anaphases, micronuclei formation, and heightened DNA damage sensitivity [50, 54], while mutant *nse2* mice display heightened levels of spontaneous sister chromatid exchange (SCE) [21]. Chromosome breaks, along with defective progression of replication forks in *nse1* mutant cells, are the most likely contributors to the higher levels of endogenous DNA damage, which are detected as increased CHK2 and γH2AX phosphorylation, as previously described for other Smc5/6 mutants in human and murine cells [26, 39]. Despite the activation of checkpoint responses, a substantial proportion of cells in the culture must resolve DNA damage to remain viable and complete cell division even in the absence of the Smc5/6 complex. From this point of view, *nse1* mutants must represent the chronification of a pathological state of chromosome non-disjunction.

### Smc5/6 and FANCM independently contribute to fork progression and genome integrity in human cells

Genome instability frequently stems from faulty replication, being an important factor driving cancer development. Mutations in the FANCM gene are associated with Fanconi anemia (FA), a rare genetic disease characterized by defects in development, bone marrow failure and cancer predisposition [55]. Mutations in the Smc5/6 complex have also been recently linked to cancer [56]. At the molecular level, FANCM and its homologs in budding and fission yeast (Mph1 and Fml1, respectively) can catalyze the reversal of replication forks in vitro [57, 58]. Interestingly, Smc5 can directly bind to and inhibit the fork reversal activity of Mph1 in vitro [34]. In fact, both the lethality and the accumulation of X-shaped junctions in yeast *smc5/6* mutants can be rescued by inactivation of Mph1 [32]. The severe depletion of Smc5/6 proteins makes *nse1-RING* mutant cells an attractive tool to study the role of Smc5/6 in DNA replication and the possible connections with FANCM regulation. However, our results indicate that the functional interaction between Smc5/6 and Mph1 is not conserved in humans. The inactivation of FANCM does not only fail to alleviate the growth of Smc5/6 mutant cells but worsens the genomic instability in human cells, leading to a strong synthetic sick phenotype and indicating that they operate in different pathways (Fig. 6E). Our observations do not exclude that FANCM and Smc5/6 work together under other circumstances, particularly at specific types of DNA lesions. For example, both are recruited to inter-strand crosslinks [59] and collaborate in their repair [60] and FANCM recruitment to stressed replication forks is partially dependent on Smc5/6 [38].

Our results also indicate that both Smc5/6 and FANCM may independently control fork speed. Smc5/6 depletion slows forks in HEK293T (Fig. 5). Although conditional depletion of Smc5/6 subunits was previously reported not to affect fork rate in HCT116 cells [26], we also consistently observed a reduction in fork rates in these cells upon Smc5/6 inactivation (Supplementary Fig. 5E-F). In accordance, previous results in human cells or mouse neuronal precursors deficient in Smc5/6 function have also shown reduced fork rates and impaired restart of stalled replication forks [39, 40]. While we currently do not fully understand how Smc5/6 impinges on fork rate, a recent report suggests that Smc5/6 might prevent excessive MRE11-dependent resection, at least at stalled forks [38]. Curiously, forks do not seem to be affected by downregulation of NSE2 [21, 46], most probably because depletion of NSE2 does not destabilize the Smc5/6 complex, maybe retaining its ability to regulate replication forks. FANCM has also been shown to play a role in DNA replication under normal conditions and in response to genotoxic stress [61, 62]. We think that the synthetic sick interaction between FANCM and Smc5/6 stems from their independent roles at forks. Based on our findings, we propose that the separate roles of FANCM and Smc5/6 at forks increase the genomic instability in double mutants. In addition to its roles at forks, FANCM also participates in the maintenance of genome integrity through interactions with various partners, including the BTR complex, MHF1/2 and PCNA [33]. Since many of these factors are present at forks and have been connected to Smc5/6 [2], it would be interesting to analyze if Smc5/6 also operates in parallel to them.

Finally, the observation that the Smc5/6 complex controls fork progression in a FANCM-independent manner in humans opens the question about the underlying mechanism. Based on the known relation between Smc5/6 and DNA topology [29, 63], we speculate that the Smc5/6 complex could control fork progression by organizing chromatin fibers during DNA replication. In turn, the Smc5/6-dependent superhelical changes could alter the presence of remodelers at forks, affecting fork structure and progression. In addition, since the Smc5/6 complex bears two E3 activities able to modify other proteins, it is also possible that it controls forks progression through SUMOylation and/or ubiquitination. Further examination of the Smc5/6-dependent recruitment of fork remodelers and their post-translational modifications should help to identify downstream effectors of this vital SMC complex during DNA replication.

## Methods

### Cell lines

Human embryonic kidney (HEK293T) cell line was purchased from the American Type Culture Collection (ATCC, USA). SV-40 immortalized human lung fibroblast MRC5-VI cells were a gift from Prof Alan Lehmann, Genome Damage and Stability Centre, University of Sussex, UK. HCT116 CMV-OsTir1 SMC6-mAID NSE4A-mAID cells were kindly shared by Dr. Masato Kanemaki, National Institute of Genetics, Japan, and Dr. Ian Hickson, Center for Chromosome Stability and Center for Healthy Aging, University of Copenhagen, Denmark. SV-40 immortalized human fibroblasts, originally derived from a patient, carrying a *FANCM* homozygous mutation c.1506_1507insTA; p.Ile503* (FANCM knockout (KO) cells) were a gift from Prof. Jordi Surrallés, Department of Genetics and Microbiology Autonomous University of Barcelona, Cerdanyola del Vallès (UAB), Spain. FANCM gene expression was re-established by transduction of a lentiviral vector expressing wild type FANCM using Tet-ON 3G expression system. HEK293T, human fibroblast and MRC5-VI cells were cultured in complete Dulbecco’s modified Eagle’s medium - DMEM (Gibco) (11,594,486, Fisher Scientific) supplemented with 10% fetal bovine serum (FBS), 20 U/mL penicillin and 20 µg/mL streptomycin. To induce the expression of *FANCM* in FANCM KO cells, the antibiotic doxycycline (Dox) at a final concentration of 1 µg/mL was added to the culture medium. All cell lines were mycoplasma-negative and were grown in thermostat at 37^o^C and 5% CO_2_. Cultures were tested for mycoplasma by PCR every two weeks and cells that tested positive were discarded.

### Genome edition by CRISPR/Cas9

Edition of the human HEK293T cell line was performed by CRISPR/Cas9. A single guide RNA (sgRNA) targeting exon 7 on the *NSE1* locus (GTGGGATCAGGATGCACTTAC), was designed using Benchling CRISPR-sgRNA design tool (https://www.benchling.com). Chimeric sgRNA was cloned into plasmid pX458 (#48,138, Addgene), also expressing Cas9 endonuclease and eGFP. To generate the deletion of the RING domain of NSE1, cells at 70–80% confluence were transfected with 1 µg of the CRISPR plasmid, using Lipofectamine 2000 (Invitrogen, 11,668,019). To create the point mutation, a specific single-stranded oligodeoxynucleotide (ssODN) was used as a donor DNA, carrying the desired point mutation (c.[618_620delinsAGC], p.[Cys207Ala]), designed according to IDT (Integrated DNA Technologies, USA) instructions. Cells were nucleofected with 2.5 µg of the CRISPR plasmid and the ssODN at 0.5 µM, using Nucleofector Solution V (Lonza) and program A-023 in Nucleofector I (Amaxa, Lonza).

Single clones were isolated 24 h after transfection. Cells were diluted and distributed in 96-well plates at 0.5 cells/100 µL. Individual clones were analyzed by the amplification of the genomic region surrounding the Cas9 cleavage site by PCR (primers CGGAGTTTCTGGGACAAAGTGC and GCAGAGTTAGCCCCAGTTCAGA). Later, Surveyor Assay with T7 Endonuclease I (M302, NEB) was performed. Briefly, samples were denatured and re-annealed. After re-annealing, heteroduplexes were digested with T7 Endonuclease I and InDels were detected by positive cleavage and electrophoresis. To detect incorporated point mutations on *NSE1* RING sequence, PCR products were restricted with PvuII enzyme and mutations were detected by positive cleavage and electrophoresis. Positive clones were further analyzed by sequencing and western blot, and two clones for *nse1-ΔR* and two clones for *nse1-CA*, each carrying the desired mutations, were selected for further analysis. All clones exhibited slower growth and lacked expression of the NSE1 protein. Since both *nse1-ΔR* and *nse1-CA* clones displayed identical phenotypes, we proceeded with experiments using one clone from each mutation.

CRISPR/Cas9 gene editing of MRC5-VI cell line was performed using gRNA (CCGTAGATAAGTTGGAGGAC) targeting exon 3 of NSE1, cloned into the GeneArt CRISPR nuclease (CD4 reporter) vector (A21175, Life Technologies). 24 h after transfection with the CRISPR plasmid, cells expressing the reporter were enriched using CD4 magnetic beads (11331D, Life Technologies), then plated in 96-well plates at 0.5 cells/100 µL. Individual clones were analyzed by western blotting and sequencing.

### Western blot

Proteins were extracted with lysis buffer (2% SDS, 125 mM Tris-HCl pH 6.8). Total protein concentration was measured using DC (detergent compatible) colorimetric protein assay kit (5,000,111, Bio-Rad), according to the manufacturer’s instructions. Loading buffer (5% sucrose, 4% β-mercaptoethanol and 0.0025% bromophenol blue) was added to samples and proteins were denatured at 95ºC for 5 min. 30–100 µg protein were loaded onto Tris-Glycine gels for SDS-PAGE electrophoresis with Running Buffer (25 mM Tris, 1.44% glycine and 0.1% SDS). Proteins were transferred onto polyvinylidene fluoride (PVDF) membranes (15,239,814, Fisher Scientific), activated with methanol and equilibrated with transfer buffer (192 mM glycine, 25 mM Tris-HCl, 10 or 20% ethanol). Protein transferring was performed at 60 mA/gel for 1 h by semi-dry blotting procedure using Semi-Dry transfer system TE 77 (Pharmacia Biotech). Membranes were blocked with 5% non-fat milk in PBS-0.1% Tween-20 (PBST) for 1 h and incubated overnight with primary antibodies diluted in 0.25% milk in PBST, at 4^o^C. Membranes were washed three times with PBST and incubated for 1 h at room temperature with the secondary antibody diluted in 0.025% milk. After three washes with PBST for 10 min, membranes were incubated with Immobilon Western Chemiluminescent HRP Substrate (11,556,345, Millipore) for 5 min at room temperature protected from light. For FANCM detection, after overnight incubation with the primary antibody, membranes were washed with PBST and incubated for 1 h with a secondary antibody conjugated to biotin (Donkey anti-mouse IgG (H + L), 1:10.000, 15,343,866, Fisher Scientific). Membranes were washed three times with PBST and incubated for 1 h with Poly-HRP Streptavidin (1:30.000, 11,801,284, Fisher Scientific). Three extra washes with PBST were performed before incubation with HRP substrate. Proteins were detected by ChemiDoc XRS Imaging System (Bio-Rad) and Image Lab Software 4.0.1 (Bio-Rad).

The following primary antibodies were used: Rabbit polyclonal anti-NSE1 (1:1000), Mouse monoclonal anti-MMS21 (NSE2) (215 C) (1:1000, ab241564, Abcam), Rabbit polyclonal anti-NSE3 (1:1000), Rabbit polyclonal anti-NSE4A (1:1000, HPA037459, Sigma-Aldrich), Rabbit polyclonal anti-SMC5 (1:1000), Rabbit polyclonal anti-SMC6 (1:1000), Mouse monoclonal anti-Actin (C4) (1:1000, MAB1501, Sigma-Aldrich), Mouse monoclonal anti-GAPDH-HRP (71.1) (1:1000, G9295, Sigma-Aldrich), Mouse monoclonal anti-phospho-Histone H2A.X (Ser139) (JBW301) (1:500, 05-636, Millipore Sigma), Mouse monoclonal anti-CHK1 (G-4) (1:500, sc-8408, Santa Cruz Biotechnology), Rabbit polyclonal anti-CHK2 (1:500, 2662, Cell Signaling), Rabbit polyclonal anti-Phospho CHK1 (Ser345) (1:500, 2341, Cell Signaling), Rabbit polyclonal anti-Phospho CHK2 (Thr68) (1:500, 2661, Cell Signaling), Mouse monoclonal anti-FANCM (CV5.1) (1:100, MABC545, Sigma-Aldrich), Mouse monoclonal anti-VINCULIN (VIN-11-5) (1:1000, V4505, Sigma-Aldrich), rabbit anti-SMC1 (kindly provided by Ana Losada). Secondary antibodies used: Sheep monoclonal Anti-mouse IgG-HRP conjugated (1:10.000, NXA931V, GE Healthcare), Goat monoclonal Anti-Rabbit IgG-HRP conjugated (1:10.000, RPN4301, Fisher Scientific). Antibodies against human NSE1, NSE3, SMC5 and SMC6 were raised by immunizing rabbits with the recombinant His-tagged proteins expressed in E. coli and purified with Ni-NTA (Qiagen) and containing the full-length NSE1, NSE3 and the amino acids 600–950 of human SMC5 and 350–670 of human SMC6. In Figs. 1G and 4D, we used mouse monoclonal anti-CHK1 (E250) (1:1000, 2G1D5, Cell Signaling), mouse monoclonal anti-p53 (1C12) (1:2000, 2524, Cell Signaling), mouse monoclonal anti-phospho-p53 (Ser15) (16G8) (1:1000, 9286, Cell Signaling), rabbit polyclonal anti-Histone H3 (1:2000, ab1791, Abcam), mouse monoclonal anti-GAPDH (6C5) (1:4000, ab8245, Abcam) and rabbit polyclonal anti-SMC5, SMC6, NSE1, NSE2, NSE3, NSE4 (1:1000, Alan Lehmann’s Lab).

### Immunoprecipitation

SMC5 immunoprecipitation was performed using a rabbit polyclonal anti-SMC5 (2.5 µg per IP, NB100-469, Novus Biologicals), bound to Dynabeads Protein A (10001D, Thermofisher). A normal rabbit IgG was used in the negative control. Cell extracts were obtained in lysis buffer (20mM TRIS-HCl pH 7.5, 150 mM NaCl, 0.1% TRITON X-100, 0.5 mM DTT, 1mM PMSF, 10mM NEM, 1x protease inhibitors cocktail (05056489001, Roche)) by mechanical disruption of the cells using a 25 Gauge needle attached to a syringe, homogenizing extracts 15 times. Samples were maintained in ice for 30 min. Extracts were centrifuged at 4ºC, at 14,000 g for 15 min. Soluble cell extracts (Inputs) were incubated over night at 4ºC with the antibody pre-bound to magnetic beads. Beads were washed 4 times with lysis buffer and eluted in 30 µl of denaturing buffer (2% SDS, 125 mM TRIS-HCl pH6.8) during 10 min at 70ºC. 4x loading buffer (5% sucrose, 4% β-mercaptoethanol and 0.0025% bromophenol blue) was added to samples to a final 1x concentration, and proteins were denatured at 95ºC for 5 min, before SDS-PAGE. Analysis of the different subunits of the SMC5/6 complex were performed using antibodies previously described in Western blot section.

### Drugs and cell treatments

The following drugs were used throughout the study: MG132 [10 µM] (SML1135, Sigma-Aldrich), proteasome inhibitor; MLN4924 [1 µM] (505,477, Millipore Sigma), NEDD8 pathway inhibitor; MMS (Methyl methanesulfonate) [250 µM] (129,925, Sigma-Aldrich), DNA alkylating agent; Concanamycin A [50 nM] (sc-202,111, SantaCruz Biotechnology), lysosomal degradation inhibitor; chloroquine [100 µM] (C6628, Sigma-Aldrich), autophagy inhibitor. Treatments were performed as indicated.

### Transfection

Transfection experiments in HEK293T cells were performed using polyethylenimine (PEI) (408,727, Sigma). Cells were seeded at 60–80% confluence. After 24 h, cells were washed with Opti-MEM I reduced serum media (31,985,070, Fisher Scientific) and incubated for 1 h with the plasmid DNA and PEI mixture in a ratio 1 µg:10 µL, diluted in Opti-MEM. Later, Opti-MEM was replaced with DMEM and cells were incubated overnight for overexpression of the target gene. Human *NSE1* coding region was cloned into pEGFP-C1 for transient expression. Point mutations were created by site directed mutagenesis.

For MRC-VI, cells were seeded and grown for 24 h to 60–70% confluence. Transfection mixture (1 µg plasmid DNA: 2 µl Turbofect transfection reagent (Thermo Fisher Scientific): 100 µl serum-free medium was incubated for 15 min before addition to the cells. For rescue experiments, the NSE1 ORF was subcloned into pCI-Neo vector (Promega).

Transient gene depletion was carried out using the Lipofectamine RNAiMax transfection reagent (12,323,563, Fisher Scientific), according to the manufacturer’s instructions. The AAGCUCAUAAAGCUCUCGGAAdTdT human FANCM siRNA was used at a final concentration of 20 nM [64].

### Virus production, infection and selection

For generation of stable cell lines, lentiviral particles were produced by co-transfection of cells with PEI, in Opti-MEM, in a 1:1:2 ratio with envelope plasmid pVSV-G, packaging plasmid pMDLg/pRRE and lentiviral vector: pLKO1-Puro-Scramble shRNA (SIGMA, SHC002), pLKO1-Puro-shRNA FANCM (CAAACCATGTTCACAATTAGA [65], pLKO1-Puro-shRNA SMC5 (SIGMA, TRCN0000147918). After 48 h, the lentiviruses were harvested, filtered through a 0.45 μm syringe filter and stored at -80ºC. For the infection, cells were seeded at 60% confluence and infected with lentivirus using polybrene (TR-1003-G, Milipore Sigma) at a final concentration of 8 µg/mL. After 24 h, the medium was changed and after 48 h, cells were split and selected for 5–10 days with neomycin or puromycin at a concentration of 800 µg/mL or 1 µg/mL, respectively. The overexpression or downregulation efficiency was detected by Western blot analysis.

### In vivo fluorescence microscopy

Cells transfected with GFP-expressing vectors were analyzed under fluorescence microscopy. Images were acquired with an Olympus IX71 microscope, with a 20x/0.40 objective. In each condition, 150 individual cells were analyzed, intensity ratio of nucleus/cytoplasm was measured using ImageJ Software (National Institute of Health).

### Cell proliferation analysis

For analysis of proliferation rate and cell viability, HEK293T cells were seeded at a concentration of 10^5^ cells/mL (only viable cells) in 6 well plate. The cell proliferation was followed for 3 days and analyzed under light microscopy using a trypan blue (T8154, Sigma-Aldrich) exclusion assay. After trypsinization and centrifugation at 1000 rpm for 5 min, cell suspensions were diluted with 0.4% trypan blue solution in a ratio of 1:1, and incubated for 5 min. Cells were counted with a haemocytometer counting chamber after 24 h, 48 h and 72 h. The growth curve graphs are representative of three independent experiments.

To determine proliferation rate of MRC5-VI cells and derivatives, cells were seeded at 5·10^4^ cells/ml in 96-well plates. Cell proliferation was followed over 4 days using the WST-1 Cell Proliferation Assay kit (Abcam), according to the manufacturer’s instructions. Growth curves show relative absorbance (480 nm) and are representative of three independent experiments.

### Time-lapse microscopy

To assess cell cycle progression by time-lapse video microscopy, wild type HEK293T at a concentration of 6·10^4^ cells, and NSE1 mutants with a concentration of 8 ·10^4^ cells, were plated in 35 μm µ-dishes (81,156, Ibidi) coated with collagen (100 µg/mL). After 72 h, cells were monitored by confocal microscopy (Olympus FV1000), equipped with 37ºC and 5% CO_2_ chamber. Six fields were examined for each cell line, with images acquired every 10 min. Mitosis duration was measured based on the specific morphological characteristics of mitosis and quantified by using ImageJ Software (NIH, USA).

### Cell cycle analysis by flow cytometry (FACS)

For cell cycle analysis by DNA content measurement, 1·10^6^ cells were washed twice with PBS and fixed with cold 70% ethanol for 24 h. After washing twice with PBS, cells were centrifuged, and DNA content was stained by incubating pelleted cells with 50 µg/mL propidium iodide (PI) (P-4170, Sigma-Aldrich) and 50 µg/mL RNase A (R6513, Sigma-Aldrich) diluted in PBS for 15 min at 37ºC. Samples were analyzed by Flow Cytometer FACS - Canto II (Becton Dickinson).

For double-staining flow cytometry exponentially growing cells (80% confluence) were treated or not with 5-Bromo-2’-deoxyuridine (BrdU) (B5002, Sigma-Aldrich) at a concentration of 10 µM for 30 min at 37^o^C. Cells were harvested, along with the supernatant, centrifuged and fixed with 70% cold ethanol for 24 h at -20^o^C. After centrifugation and washing with PBS-Tween-20 0.05% (PBST) to remove the traces of ethanol, pelleted cells were treated with 2 M (2.16 M) HCl with 0.1% Triton X-100 for DNA denaturation and incubated for 15 min at RT. HCl was neutralized with 100 mM Na_2_B_4_O_7_ (pH 8.5). After centrifugation, cells were washed with PBS-T and centrifuged again. The obtained pellets were resuspended and blocked with 3% BSA with PBST for 45 min. Next, after centrifugation, pelleted cells were incubated with anti-BrdU (ab6326, Abcam; 1:250) and anti-MPM-2 (05-368, Millipore, 1:250) antibodies for 1 h at RT. Samples were washed with PBST, spin for 2 min at 3000 rpm and incubated with secondary Alexa Fluor 647 anti-mouse (A31571, Invitrogen, 1:500) and Alexa Fluor 488 anti-rat (A21208, Invitrogen, 1:400), respectively, for 45 min in the dark. Cell pellets were washed with PBST and stained with PI (1% PI with 0.1 µg/µL RNase in PBS) and then subjected to flow cytometry analysis for measuring total DNA content (PI), S phase (BrdU) and mitotic cells (MPM-2). Results are representative of three repeats.

### Aberrant anaphases and micronuclei

To analyze the number of micronuclei and aberrant anaphases (including chromosome bridges and lagging chromosomes), replicating cells were washed once with PBS, then pre-extracted during 1 min with PME buffer (20 mM PIPES-NaOH pH 6.8, 10 mM EGTA, 1 mM MgCl_2_ in water). Cells were fixed with formaldehyde 3.7% for 10 min at room temperature, washed three times with PBS and permeabilized with 0.15% Triton X-100 in PBS for 10 min at room temperature. Cells were washed once with PBS before Hoechst staining for 15 min at room temperature. Slides were mounted with Mowiol (81,381, Sigma-Aldrich) and dried overnight before visualization. Images were acquired with an Olympus BX51 microscope, with a 60x/1.42 oil immersion objective. In each condition, 80 individual anaphases or micronuclei were analyzed.

### Sister chromatid exchange assay (SCE)

For visualization of SCE, HEK293T (wild type) and NSE1-ΔR mutants were incubated with BrdU (B5002, Sigma) at a concentration of 50 µM for two cell cycles. The duration of this treatment depends on the proliferation rate of tested cell lines (36 h for HEK293T and 72 h for NSE1-ΔR mutants, respectively). Cells were blocked at metaphase with KaryoMAX Colcemid (0.05 µg/mL) (15,212,012, Thermo-Fisher Scientific) for 2 h. After washing with PBS, cells were trypsinized, centrifuged and exposed to hypotonic shock with 0.03 M sodium citrate for 25 min at 37^o^C. Chromosomes were fixed with a solution of methanol: acetic acid in a ratio of 3:1 and metaphase spreads were prepared by releasing a drop onto a glass slide. Metaphase chromosome spreads were stained with 0.1 mg/mL acridine orange (A6014, Sigma-Aldrich) and incubated for 5 min at RT. The stained slides were carefully rinsed with distilled water, incubated for 1 min in Sorenson buffer pH 6.8 (0.1 M Na_2_HPO_4_, 0.1 M NaH_2_PO_4_), mounted with Mowiol and immediately observed under fluorescent microscope Olympus BX51. The number of reciprocal exchange events per chromosome was quantified. More than 2000 chromosomes were counted in each sample. SCE events were quantified using ImageJ software.

### Clonogenic assay

Confluent cells were treated with 250 µM MMS for 2 h or left untreated. After removing the drug, cells were trypsinized, counted and seeded at 5 · 10^2^ cells/well density in a 12 well plate and let them grow. After 10 days, cells were fixed with 10% formaldehyde in PBS for 30 min at room temperature. Then, cells were stained with 0.01% crystal violet in water for 30 min. The dye mixture was removed, plates were rinsed three times with water, and the number of colonies were counted after drying. The analysis was performed using the ColonyArea plugin from ImageJ Software (National Institute of Health). Data was pooled from three independent experiments.

### DNA fiber analysis

Exponentially growing cells were pulse-labelled with 50 µM CIdU (30 min; Sigma-Aldrich, C6891), washed three times with PBS, then pulse labelled with 250 µM IdU (30 min; Sigma-Aldrich, I7125). Labelled cells were collected and mixed with unlabelled cells (in a 1:1 ratio). 2 µL of cells (1500 cells/µL) were placed in a microscope slide and mixed with 10 µL of lysis buffer (0.5% SDS, 200 mM Tris-HCl, pH 7.4, 50 mM EDTA in water). After 7 min incubation at room temperature in a humidity chamber, slides were tilted at a 10–15° angle to spread the fibers at a constant, low speed. After air drying for 20 min at room temperature, DNA was fixed onto the slides with a freshly prepared solution of methanol: glacial acetic acid at 3:1 for 5 min, then the slides were air dried completely.

For immuno-detection of labelled tracks, DNA was washed with PBS, then denatured with 2.5 M HCl for 1 h at room temperature. Slides were washed with PBS four times and blocked during 1 h with blocking buffer (1% BSA, 0.1% Tween20-containing PBS solution). DNA fibers were incubated with primary antibodies (for CldU, rat monoclonal anti-BrdU, BU1/75 (ICR1) 1:100, Abcam #ab6326; for IdU, mouse monoclonal anti-BrdU, 1:100, BD Bioscience #347,580) for 1 h at room temperature in a humidity chamber. Then, slides were washed once with 0.1% Tween20-containing PBS and twice with PBS. Slides were fixed with 4% PFA-containing PBS for 10 min at room temperature, washed three times with PBS and once with blocking buffer. Then, slides were incubated with secondary antibodies (Goat anti-rat IgG Alexa Fluor 594, 1:300, Fisher Scientific #10,348,312; Goat anti-mouse IgG1 Alexa Fluor 488, 1:300, Fisher Scientific #10,727,464) for 1 h at room temperature in a humidity chamber. Slides were washed five times with PBS, air dry and mounted with Prolong Gold Antifade Reagent (Fisher Scientific, P36930). Images were acquired with Zeiss Axioobserver Z.1 microscope, ZEN3.7 software, with either a 63x/1.4 or 100x/1.4 oil immersion objective. In each assay, 300 individual tracks were measured to estimate the fork rate. Only DNA tracks labelled with IdU, characterized by contiguous IdU-CldU signals, were analyzed to measure replication fork progression. The length of each tract was measured manually using ImageJ software (National Institute of Health). The pixel values were converted into micrometers using the microscope software scale bar. The size distribution of tract lengths was plotted as scatter dot plot with the line representing the median. Data was pooled from three independent experiments.

#### Quantification and statistical analysis

Statistical analysis was performed using Prism 8 (GraphPad Software). Details of the individual statistical tests are indicated in the figure legends and results. In all cases: ns, non-significant; *p < 0.05, **p < 0.01, ***p < 0.001, ****p < 0.0001. All experiments were repeated at least three times unless otherwise noted.

### Electronic supplementary material

Below is the link to the electronic supplementary material.

Supplementary Material 1

## Data Availability

All data required to evaluate the conclusions in the paper are present in the manuscript and the supplementary materials.
