# Supplementary material for: Crucial role of the NSE1 RING domain in Smc5/6 stability and FANCM-independent fork progression
Source: Cell Mol Life Sci. 2024 Jun 7;81(1):251. doi: 10.1007/s00018-024-05275-3 (PMC11335289; doi:10.1007/s00018-024-05275-3)
Supplement: Supplementary file 1 — Supplementary Material 1 [file 18_2024_5275_MOESM1_ESM.docx]

**Lorite et al. Supplementary Figures.**

**
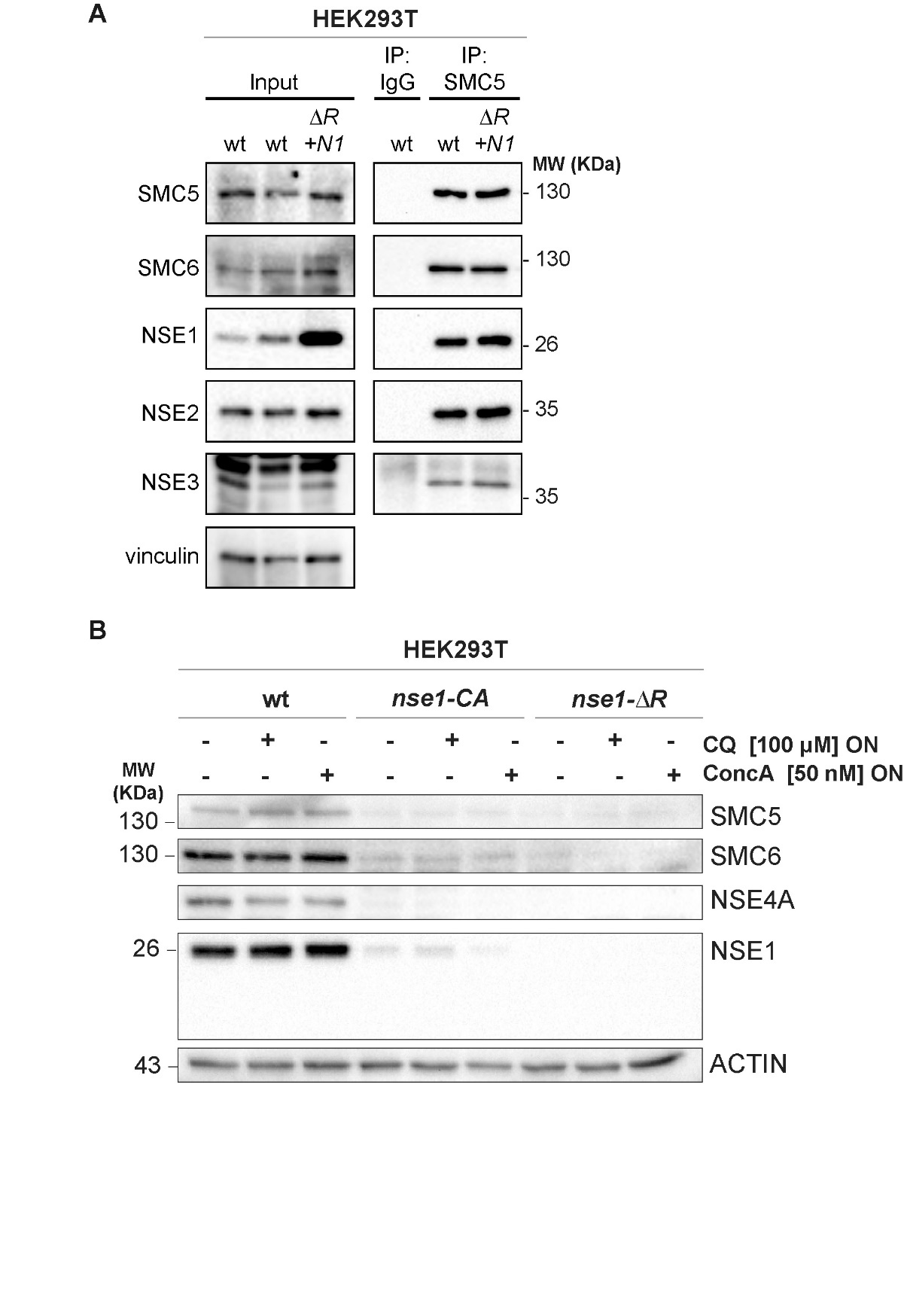
**

**Supplementary Figure 1. NSE1 RING mutants destabilize the Smc5/6 complex A.** Re-expression of NSE1 rescues Smc5/6 complex integrity in *nse1-ΔR* cells. Protein extracts from wild type cells (wt) or *nse1-ΔR* cells rescued with wild type NSE1 (Δ*R*+N1) HEK293T cells were subjected to immunoprecipitation with mock antibodies (IgG) or anti-SMC5 as indicated. Inputs and immunoprecipitates (IP) were analyzed by western blot using the indicated antibodies. **B.** Inhibition of autophagy or lysosomal degradation does not affect the expression of NSE1 RING mutant proteins. Western blot analysis of HEK293T wild type (wt), *nse1-ΔR* or *nse1-CA* cells treated (+) or not (-) with the autophagy inhibitor chloroquine (CQ) or the lysosomal degradation inhibitor concanamycin A (ConcA) at the indicated concentrations. Cells were treated overnight before collection. Note that neither mutant proteins nor other Smc5/6 tested (SMC5, SMC6 and NSE4A) can be stabilized by CQ or ConcA.

**
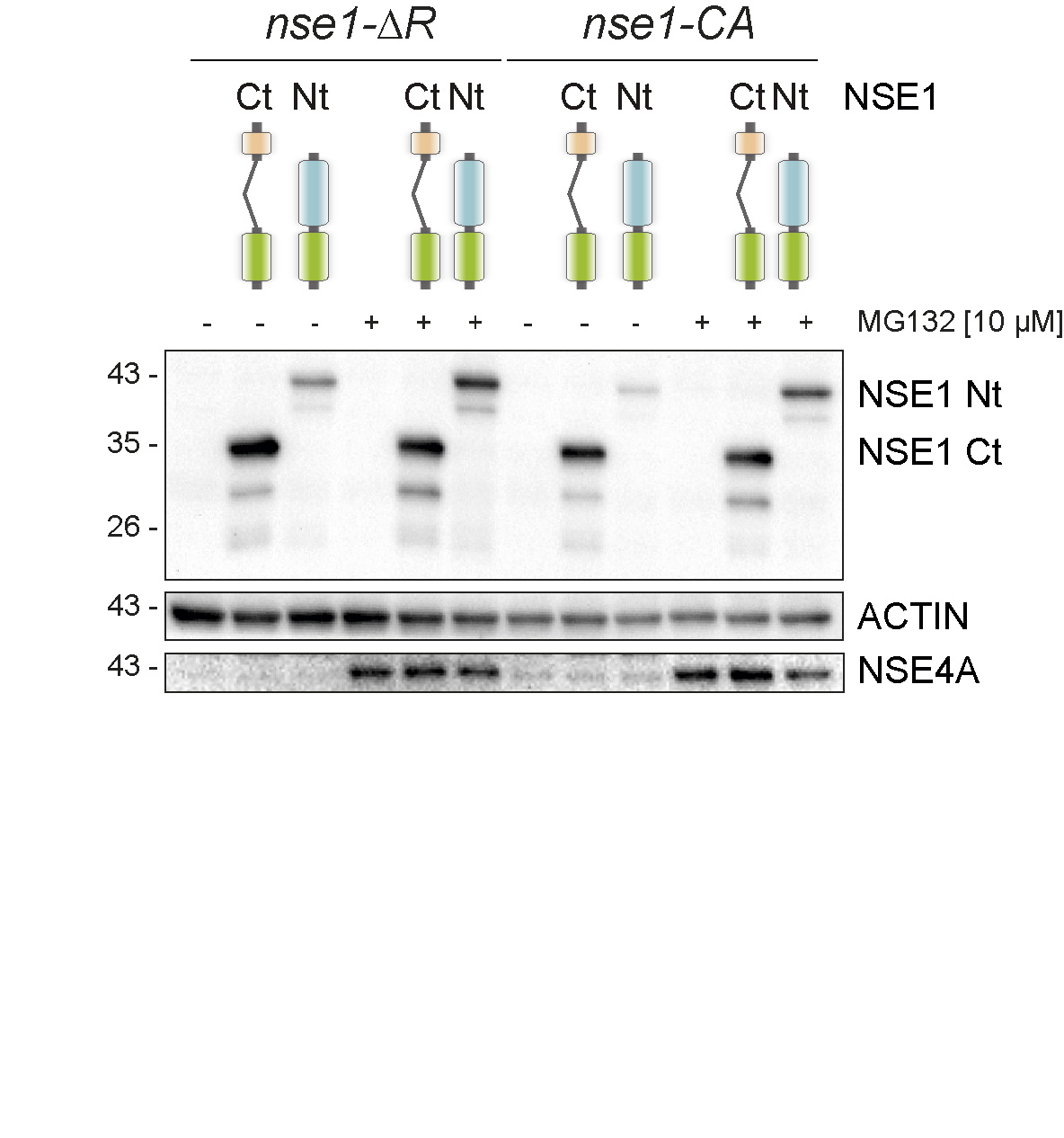
**

**Supplementary Figure 2. NSE1 proteins lacking the RING domain can be stabilized by proteasome inhibition.** Western blot analysis performed on *nse1-ΔR* or *nse1-CA* HEK293T cells expressing N-terminal GFP fusions to the either the NSE1 N-terminal domain (Nt) or the NSE1 C-terminal domain (Ct). Cells were left unchallenged (-) or treated with 10 μM MG132 for 24h (+). Note that the protein levels for the N-terminal domain can be upregulated by inhibition of the proteasome, while this effect is not detectable for the C-terminal RING domain. Proteasome inhibition also leads to stabilization of the NSE4A subunit of the Smc5/6 complex.


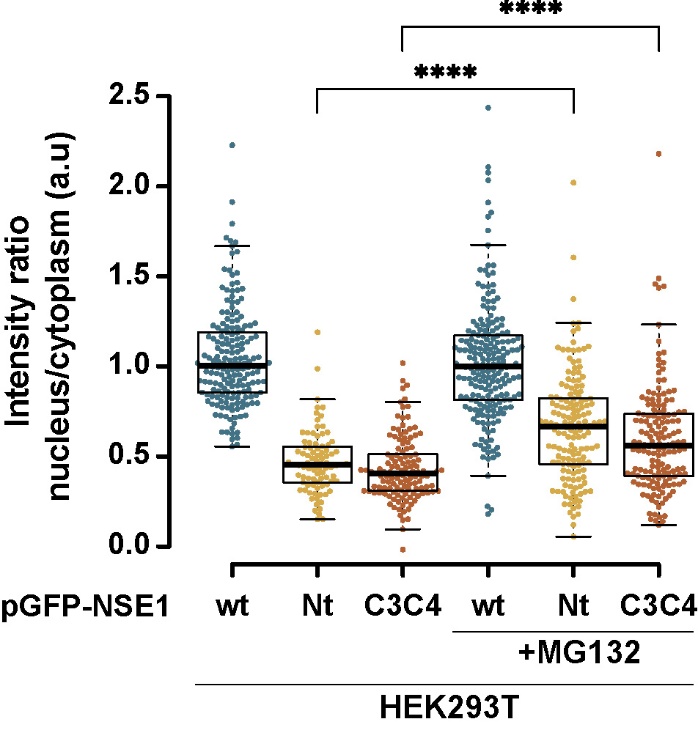


**Supplementary Figure 3. NSE1 RING mutant protein localization after proteasome degradation.** Quantification of in vivo fluorescence microscopy of HEK293T cells expressing the indicated NSE1 fusions to GFP: wild type (wt), NSE1, N-terminal domain without RING domain (Nt) or NSE1-C3C4 mutant (C3C4) fusions of NSE1. Cells were left untreated or treated with MG132 10 μM for 4 hours (+MG132). ****, P<0.0001 by a one-way ANOVA followed by Tukey’s multiple comparison test.


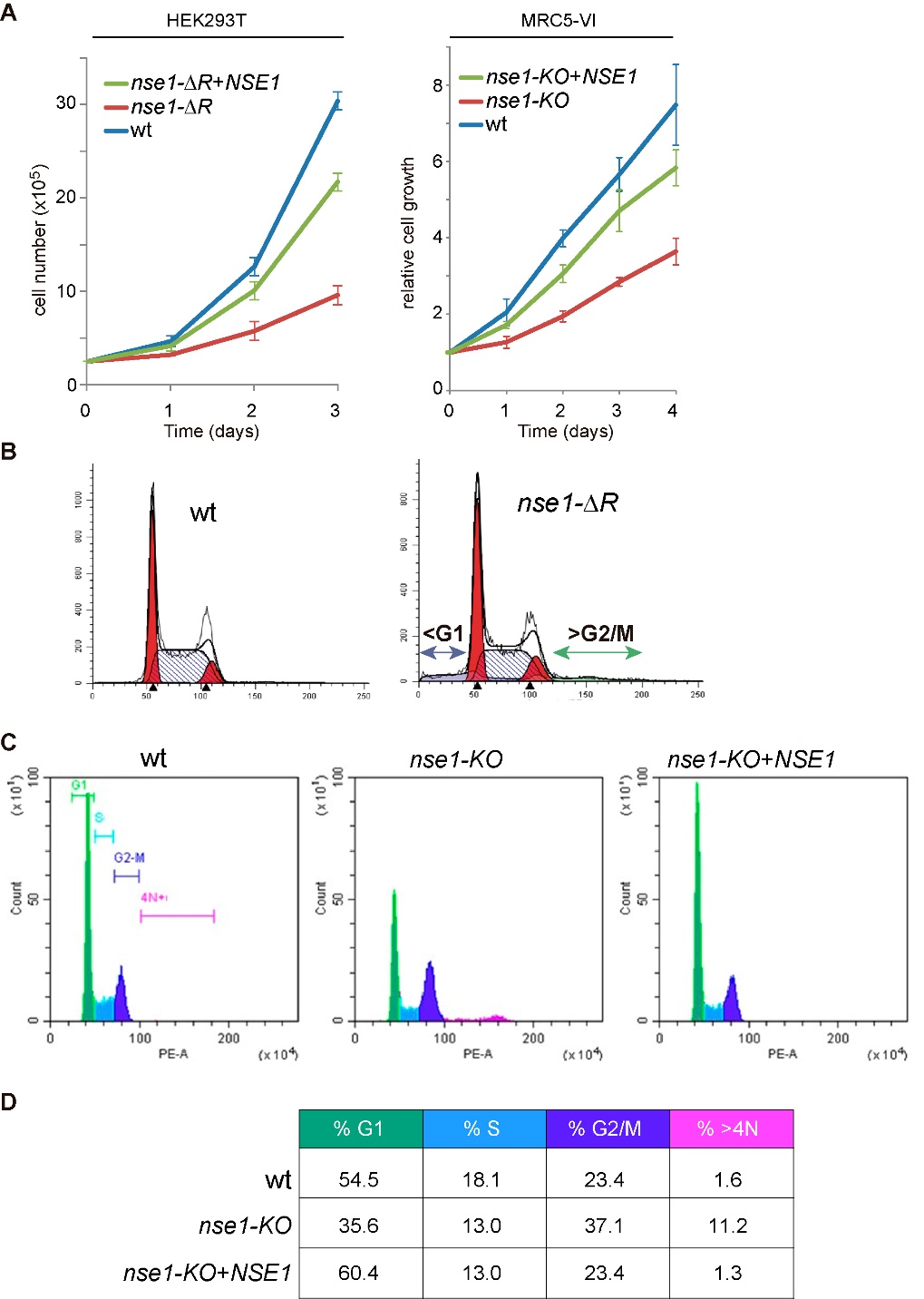


**Supplementary Figure 4. Mutations in the RING domain of NSE1 cause growth and cell cycle defects. A.** Left panel. Growth curve analysis of HEK293T wild type (wt) *nse1-ΔR* and *nse1-ΔR* cells rescued by expression of wild type *NSE1* (*nse1-ΔR+NSE1*). Proliferation was followed for 3 days using Trypan blue (TB) exclusion assay. Mean and SEM values for three independent experiments are shown. Right panel. Time course of cell proliferation for MRC5-VI wild type and *nse1-KO* cells rescued (*nse1-KO+NSE1*) or not by expression of wild type *NSE1*. Data represent the mean ± SD of three independent experiments. **B**. Propidium Iodide (PI) FACS analysis of wild type and mutant *nse1-ΔR* HEK293T cells. ModFit LT software was used to identify cells in G1 and G2/M (red), in S phase (diagonal pattern), below G1 (grey) and above G2/M (green). **C.** PI-FACS profile of MRC5-VI (wt), *nse1-KO* and *nse1-KO+NSE1*cells. **D**. Quantification of cells in G1, S, G2/M and with more than 4N DNA content from gates defined in C.


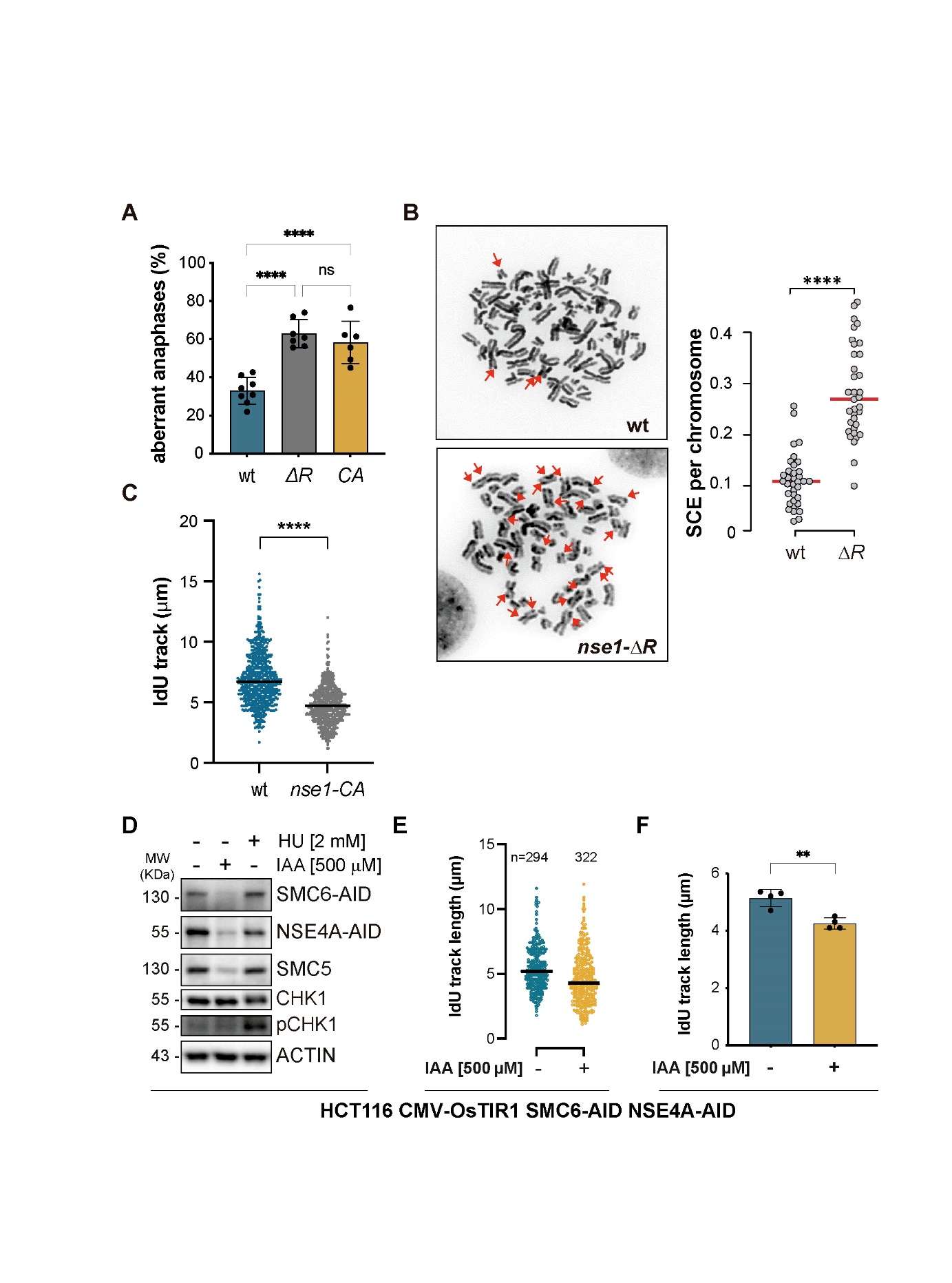


**Supplementary Figure 5. NSE1-RING and Smc5/6 mutants reduce fork speed and increase genomic instability. A.** Frequency of aberrant anaphases (anaphase bridges and lagging chromosomes) in HEK293T wild type, *nse1-ΔR* and *nse1-CA* cells, relative to the total number of anaphases scored; a minimum of 80 anaphases were counted per experiment; bars indicate means and error bars SD. ****, P<0.0001 by a one-way ANOVA followed by Tukey’s multiple comparison test; ns= non significant. **B.** Sister chromatid exchanges (SCE) were quantified in chromosome spreads of wild type (wt) and *nse1-ΔR* cells in metaphase, by BrdU and Acridine Orange staining. Left, representative images of wild type and mutant spreads, red arrows indicate chromatid exchanges; right, quantification of SCE per chromosome in wild type (wt) and *nse1-ΔR* (*ΔR*) mutant spreads. Circles represent individual metaphases, red bars are mean values. ****, P<0.0001 by one-way Anova using Bonferroni-Holm post-hoc test.  **C.** Length of the IdU tracks in DNA fibers from HEK293T cells with the indicated genotype, measured by Image J Software. Data points shown are aggregates from two independent experiments. ****, P < 0.0001 by a Kruskal-Wallis followed by Dunn’s multiple comparisons test. **D**. SMC6-AID NSE4A-AID double mutant HCT116 cells were treated (+) or not (-) with 500 μM auxin (IAA) for 4 hours and protein samples analyzed by Western blot with the indicated antibodies. As a control for checkpoint activation cells were treated with HU 2 mM for 1 hour. Note that Smc5/6 inactivation does not activate Chk1. **E**. HCT116 SMC6-AID NSE4A-AID cells, treated (+) or not (-) with 500 μM auxin (IAA) for 4 hours, were pulse-labelled with CldU for 30 minutes and with IdU for an additional 30 minutes and subjected to DNA fibers analysis. Data points represent single IdU fiber length measurements from an individual experiment after measurement. **F**. Mean IdU track length from four independent experiments in HCT116 SMC6-AID NSE4A-AID cells treated (+) or not (-) with 500 μM auxin (IAA) for 4 hours.


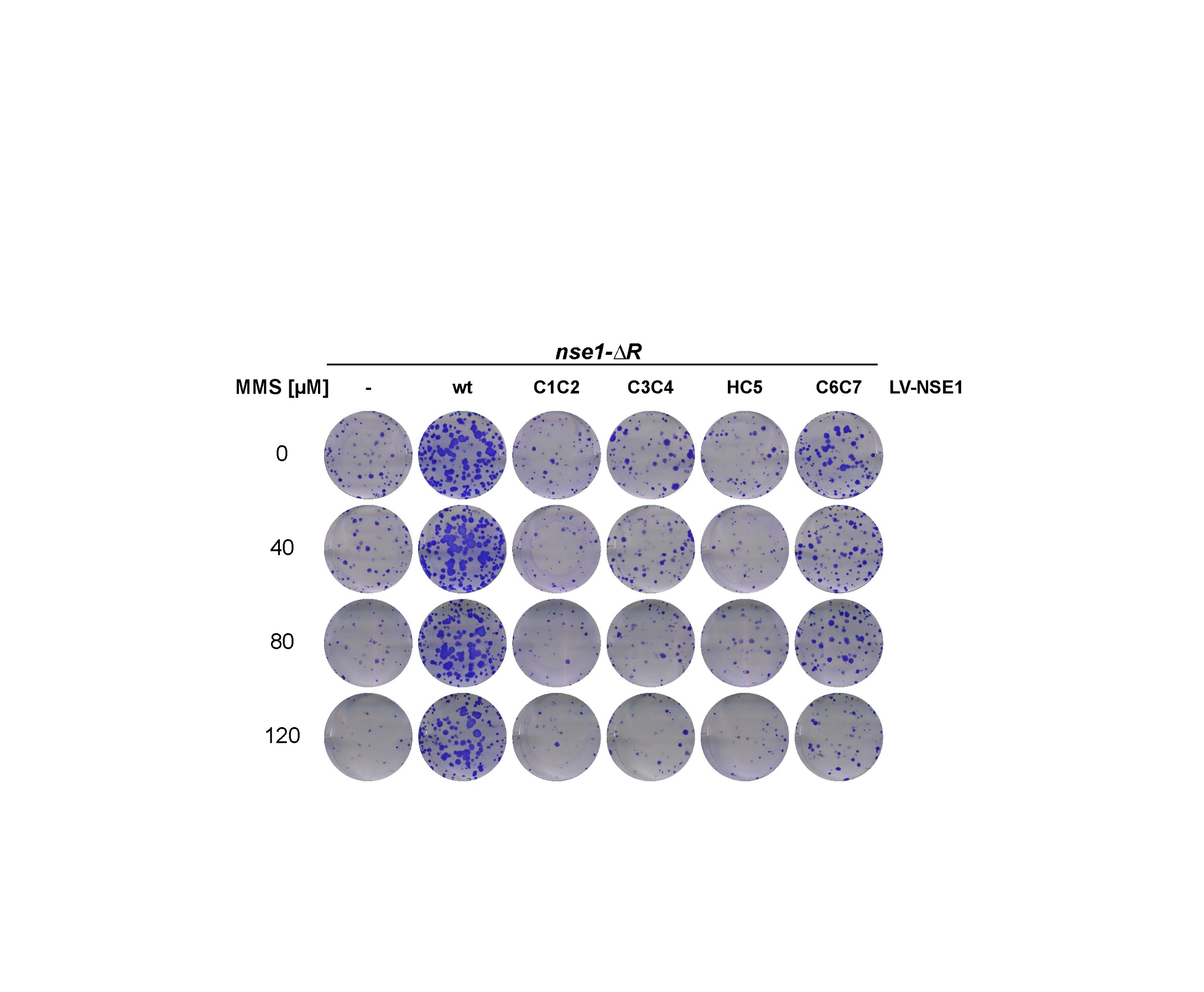


**Supplementary Figure 6. Differential contribution of the two zinc-coordinating centers in the NSE1 RING to DNA damage sensitivity.** Clonogenic assay of HEK293T *nse1-ΔR* cells infected with the indicated *NSE1*-expressing lentiviral vectors. Cells were seeded, allowed to grow for 3 days, and grown for 4 more days with the indicated concentrations of MMS.
